# Biologic use in psoriatic arthritis and ankylosing spondylitis patients: a descriptive epidemiological study using linked, routine data in Wales, UK

**DOI:** 10.1093/rap/rkab042

**Published:** 2021-06-27

**Authors:** Roxanne Cooksey, Muhammad Azizur Rahman, Jonathan Kennedy, Sinead Brophy, Ernest Choy

**Affiliations:** Division of Infection and Immunity, CREATE Centre, Section of Rheumatology, School of Medicine, Cardiff University, Cardiff; Health Data Research UK, Swansea University Medical School, Swansea; National Centre for Population Health and Wellbeing Research, Swansea, UK; Cardiff School of Technologies, Cardiff Metropolitan University, Cardiff, Wales; Health Data Research UK, Swansea University Medical School, Swansea; Health Data Research UK, Swansea University Medical School, Swansea; National Centre for Population Health and Wellbeing Research, Swansea, UK; Division of Infection and Immunity, CREATE Centre, Section of Rheumatology, School of Medicine, Cardiff University, Cardiff; National Centre for Population Health and Wellbeing Research, Swansea, UK

**Keywords:** psoriatic arthritis, ankylosing spondylitis, biologics, outcomes, treatment pathways, electronic health records

## Abstract

**Objectives:**

PsA and AS are chronic diseases associated with significant morbidities. National and international management guidelines include treatment with biologic therapies to improve outcomes and quality of life. There are limited real-world data on the patients’ journey from symptom onset to diagnosis and treatment in the UK. We use real-life, linked health data to explore patient pathways and the impact of biologics on patient outcomes.

**Methods:**

Data from the Secure Anonymised Information Linkage databank in Wales were used to assess diagnosis and treatment of patients ≥18 years of age with at least one International Classification of Diseases, Tenth Revision code present for PsA/AS in rheumatology clinic data and at least one Read code present in primary care records. We investigated the use of biologics while exploring demographics, comorbidities and surgical procedures of 641 AS patients and 1312 PsA patients.

**Results:**

AS patients were significantly younger at diagnosis and were predominantly male. The average time from presenting symptoms to diagnosis of AS and PsA was 7.9 (s.d. 5.5) and 9.3 (s.d. 5.5) years, respectively. The proportion of patients receiving biologic treatment was significantly higher in AS (46%) compared with PsA patients (28.8%); of these, 23.1% of AS and 22.2% of PsA patients stopped/switched a biologic. There was a significant reduction in primary care involvement, sick notes and disability living allowance for both AS and PsA patients following biologic initiation.

**Conclusion:**

This real-world descriptive study confirms that patients treated with biologics have reduced disability and time off work despite being initiated ∼13 years after the first symptoms and 6 years after diagnosis.

**Key messages**

- Using real-world electronic data, we found that following biologic treatment reduced primary care utilization.
- Disability-related payments and sick notes issued in primary care also decreased following treatment.
- Biologics are effective in treating AS and PsA patients despite delays in diagnosis and treatment.

## Introduction

PsA and AS are chronic inflammatory arthritic conditions associated with significant morbidity and poorer quality of life. The diagnosis of these conditions is often delayed [1, 2] and this continues to be an issue in the UK [3].

A recent study explored the patient pathway of receiving a diagnosis of AS from the patient perspective in the USA. The sample was recruited online, comprising self-reported AS-diagnosed individuals. The results demonstrated a diagnosis delay, with back pain as the presenting feature that led respondents to seek treatment. Misdiagnosis was also an issue, with diagnoses of back problems, sciatica, orthopaedic issues, osteoarthritis, psychosomatic disorders and anxiety and depression associated with a longer time to AS diagnosis. Significantly more women were misdiagnosed with fibromyalgia and psychosomatic issues compared with men [2].

Misdiagnosis and diagnosis delay of inflammatory arthritic conditions can have a substantial impact; for instance, in PsA, just a 6 month delay from symptom onset to the first visit to a rheumatologist contributed to greater peripheral joint erosion and worse function long-term [4]. Moreover, AS and PsA have a negative impact on quality of life and affect career choices and the ability to work [5–8]. Reduced work productivity, work disability and more absenteeism have been reported for both AS [9–14] and PsA [8, 15, 16]. Additionally, a delay in diagnosis means that medications used to treat the conditions are not accessed until later in the disease process, further aggravating the health and social care impact of AS and PsA.

The first line of treatment for AS and PsA is NSAIDs followed by DMARDs for PsA patients. For severe disease, the National Institute of Health and Clinical Excellence (NICE) treatment guidelines state that biologic drugs can be prescribed when these treatments have failed or are poorly tolerated [17, 18]. From clinical trial data we know that PsA and AS patients respond well to treatment with biologic agents [19, 20]. However, little is known from real-world data on the effect of these drugs on patient outcomes. In particular, the direct and indirect healthcare effect of biologic initiation during established disease is not clear. In this study we linked data from primary and secondary care to specialty data from rheumatology departments to follow the real-world patient pathway from symptom onset to diagnosis to assess the impact of AS and PsA on patients.

We explored treatments used, comorbidities and health outcomes, primary care resource utilization in the form of visits to the general practitioner (GP), productivity losses from records of sick notes issued in primary care indicating individuals are not fit to work and disability payments, which all demonstrate functional impairment.

## Patients and methods

In Wales, UK, routinely collected electronic health records were extracted and linked from the Secure Anonymised Linkage (SAIL) Databank. The SAIL Databank holds >1 billion anonymized records from >90% of the people living in Wales, which has a population of 3 million. It uses a split-file approach to ensure anonymization and overcome issues of confidentiality and disclosure in health-related data warehousing. Demographic data from primary and secondary care, as well as other data sources such as cancer registries, social care and education are sent to a partner organization, the National Health Service Wales Informatics Service, where identifiable information is removed; clinical data are sent directly to the SAIL Databank and an individual is assigned an encrypted anonymized linkage field (ALF). The ALF is used to link anonymized individuals across datasets, facilitating longitudinal analysis of an individual’s journey through multiple health, education and social datasets. Data collected by physicians in primary care are captured using Read codes (5-digit codes related to diagnosis, medication and process of care codes) [21]. Hospital inpatient and outpatient data are collected in the Patient Episode Database for Wales, which contains clinical information regarding patients’ hospital admissions, discharges, diagnoses and surgeries using the International Classification of Diseases, Tenth Revision (ICD-10) clinical coding system. Data from six rheumatology departments in South Wales are also available. The rheumatology department data also uses the ICD-10 codes and contains information pertaining to rheumatology appointments, such as medications prescribed by rheumatologists and rheumatology assessments.

Patients ≥18 years of age at diagnosis were identified from the rheumatology dataset by relevant ICD-10 codes present for AS (ICD-10: M45) and PsA (ICD-10: L40.5). The rheumatology data was linked at the patient level to primary care data where Read codes were also present for AS (N100.) and PsA (M160., M166, M1601, M1602, M160z), to ensure that patients could be followed through their primary healthcare records. Codes for AS were used in the absence of codes available for axial SpA. Primary care data provided medications taken, comorbidities and healthcare resource utilization. Data linkage to hospital admissions data allowed surgical procedures and hospitalizations to be explored. Data were included from 2009 to 2018 to coincide with optimum data coverage/biologic prescription and available data.

Data held in the SAIL Databank are anonymized and therefore no ethical approval was required. All data contained in the SAIL Databank have permission from the relevant Caldicott Guardian or Data Protection Officer. This study was approved by the SAIL Databank Information Governance Review Panel.

### Exposures of interest

Exposures of interest included the medications used to treat PsA and AS patients, including NSAIDs and DMARDs, and whether the patient was receiving biologic treatment or not.

### Outcomes

Outcomes included commencing a biologic therapy, healthcare resource utilization and receipt of UK government disability payments. Biologic treatment failure was defined as stopping/starting/switching to another biologic. A biologic treatment was assumed to stop when no further codes were present in the data and an alternative biologic treatment had codes succeeding the previous biologic. Healthcare resource utilization included visits to primary care and sick notes issued by a GP (primary care physician). UK government disability payments [Personal Independence Payments (PIP) or Disability Living Allowance (DLA)] are issued to individuals with a disability and/or health conditions when there are difficulties with daily living and mobility for >3 months, with the expectation that these will continue for at least a further 9 months.

### Covariates of interest and confounding factors

The baseline covariates considered were age, sex, BMI, level of social deprivation, disease duration, smoking and alcohol use. In addition, comorbidities were identified from Read codes present in primary care health records (cardiovascular disease, diabetes, hyperlipidaemia, hypertension, interstitial lung disease; see Supplementary Table S1, available at *Rheumatology Advances in Practice* online). ICD-10 codes for serious infections (Supplementary Table S2, available at *Rheumatology Advances in Practice* online) and National Clinical Coding Standards OPCS-4 for orthopaedic surgery (Supplementary Table S3, available at *Rheumatology Advances in Practice* online) from hospital admissions data were also included. Unfortunately, acute phase reactants, ESR and C-reactive protein measurements were unavailable at the time of analysis. For a summary of the data source for all variables, see Supplementary Table S4, available at *Rheumatology Advances in Practice* online.

### Statistical analysis

Descriptive statistics were used to examine the covariate distribution at baseline. Cox proportional hazards models were employed to calculate the hazard ratio (HR) of factors associated with initiating biologic treatment and biologic treatment failure in PsA and AS patients, controlling for potential confounders. Censoring occurred when a patient failed a biologic, was lost to follow-up (i.e. moved out of the area and longer contributed data) or died. Univariate analyses were performed to determine significance and candidate variables to be included in the Cox proportion hazards models (inclusion threshold *P* < 0.05).

## Results

### Demographics

There were 641 and 1312 patients present in the rheumatology clinic with ICD-10 codes for AS and PsA, respectively. The AS population was 24% (154/641) female, compared with 51.8% (679/1312) for PsA patients. The PsA population was significantly older [difference 3.9 (95% CI 2.6, 5.2)] and had significantly increased BMI [difference 2.5 (95% CI 1.9, 3.1)] compared with AS patients (Table 1).

**Table 1:** Characteristics of AS and PsA patients

Characteristics	AS (*n* = 641)	PsA (*n* = 1312)	Difference (95% CI)
Female, % (*n*)	24 (154)	51.8 (679)	27.7 (23.3, 31.9)^*^
BMI, mean (s.d.)	28 (5.6)	30.5 (6.7)	2.5 (1.9, 3.1)^*^
Townsend deprivation score, mean (s.d.)^†^	3.1 (1.5)	3.0 (1.5)	0.1 (−0.2, 0)
Age at diagnosis, years, mean (s.d.)	42.6 (14)	46.5 (13.7)	3.9 (2.6, 5.2)^*^
Presenting back (AS) or peripheral (PsA) pain pre-diagnosis, % (*n*)	93 (596)	88.6 (1162)	4.4 (1.7, 6.9)^*^
Time from back/peripheral pain to diagnosis, years, mean (s.d.)	7.9 (5.5)	7.7 (5.6)	0.2 (−0.3, 0.7)
Alcohol use, % (*n*)	86 (551)	87.7 (1150)	1.7 (−1.4, 5.0)
Smoker, % (*n*)	11.4 (73)	9.1 (119)	2.3 (−5.4, 0.5)
GP visits per annum, mean (s.d.)	50.6 (32.5)	58.7 (34.4)	8.1 (4.9, 11.3)^*^
Existing diabetes, % (*n*)	3.7 (24)	4.5 (59)	0.8 (−1.3, 2.5)
Existing cardiovascular disease, % (*n*)	3.7 (24)	3.3 (43)	0.4 (−2.4, 1.2)
Existing hyperlipidaemia, % (*n*)	5 (32)	6.3 (83)	1.3 (−1.0, 3.4)
Existing hypertension, % (*n*)	19.2 (123)	23.6 (309)	4.4 (0.5, 8.1)
Hospitalised for serious infections, % (*n*)	5.9 (38)	6.9 (90)	0.9 (−1.5, 3.1)
Issued disability living allowance, % (*n*)	27.5 (176)	18.3 (240)	9.2 (5.2, 13.3)^*^
Sick notes issued by GP, % (*n*)	16.2 (104)	9.6 (126)	6.6 (3.5, 10)^*^
Any use of NSAIDs in GP records, % (*n*)	96.7 (620)	94.3 (1237)	2.4 (0.4, 4.2)^*^
Any use of DMARDs in GP records, % (*n*)	41.2 (264)	82.3 (1018)	36.4 (31.9, 40.7)^*^
Prescribed a biologic, % (*n*)	46 (295)	28.8 (378)	17.2 (12.6, 21.8)^*^
Characteristics of patients administered biologics	AS (*n* = 295)	PsA (*n* = 378)	
GP involvement within 1 year pre-biologic initiation, mean (s.d.)^‡^	69.7 (143.5)	75.1 (167.4)	5.4 (−18.6, 29.4)
GP involvement within 1 year post-biologic initiation, mean (s.d.) ^‡^	8.8 (26.3)	10.7 (36.3)	1.9 (−3.0, 6.8)
GP involvement per annum, mean (s.d.) ^‡^	51.6 (31.2)	60.6 (31.8)	9 (4.2, 13.8)^*^
Visits to rheumatologist within 1 year pre-biologic, mean (s.d.)	1.0 (0.2)	1.0 (0.2)	0 (−0.0, 0.0)
Visits to rheumatologist within 1 year post-biologic, mean (s.d.)	1.0 (0.2)	1.0 (0.2)	0 (−0.0, 0.0)
NSAIDs pre-biologic use, % (*n*)	81 (239)	78.6 (297)	2.5(3.8, 8.5)^*^
Number of NSAIDs used pre-biologic, mean (s.d.)	11.3 (3.1)	11.5 (3.1)	0.2 (−0.3, 0.7)
DMARDs pre-biologic use, % (*n*)	16.9 (50)	35.7 (135)	18.8 (12.1, 25)^*^
Number of DMARDs used pre-biologic, mean (s.d.)	2.5 (1.8)	3.0 (1.5)	0.5 (0.3, 0.8)^*^
Time to biologic from diagnosis, years, mean (s.d.)	6.3 (4.8)	6.2 (4.6)	0.1 (−0.8, 0.6)
Biologic duration, years, mean (s.d.)	2.9 (2.7)	2.5 (2.7)	0.4 (−0.1, 0.8)
Biologic treatment stop/change/fail, % (*n*)	23.1 (68)	22.2 (84)	0.9 (−5.5, 7.3)

* *P* < 0.05. ^†^Where 1 = most affluent and 5 = most deprived. ^‡^Involvement includes face-to-face visit, telephone consultation, prescription and administration (request for tests, letters).

The rate of GP involvement (including visits, telephone consultations, letters, prescriptions and administrative tasks) per annum was significantly higher in the PsA patients compared with AS patients [difference 8.1 (95% CI 4.9, 11.3)], while disability-related payments [difference 9.2 (95% CI 5.2, 13.3)] and sick notes [difference 6.6 (95% CI 3.5, 10)] were significantly higher in AS patients compared with PsA patients (Table 1).

### Presenting symptoms

The majority of AS and PsA patients had back pain Read codes prior to diagnosis (93% and 94.4%, respectively). With regard to peripheral joint pain symptoms pre-diagnosis, PsA patients had a significantly higher rate of this presenting symptom compared with AS patients (88.6% and 82.7%, respectively). The average time from onset of symptoms to diagnosis was longer for those with back pain compared with those with peripheral joint symptoms [7.9 years (s.d. 5.5) and 6.1 (s.d. 5.7), respectively]. However, the reverse was true for PsA; those with peripheral joint pain were diagnosed 1.6 years earlier than those with back pain [7.7 years (s.d. 5.6) and 9.3 (s.d. 5.5), respectively] (Table 1).

### NSAID and DMARD treatment

Prior to starting biologic therapy, NSAIDs were frequently used in AS and PsA patients (95.6% and 93.7%, respectively). Many of the AS patients were treated with DMARDs prior to biologics [62.4% (184/295)] compared with the majority of the PsA patients [89.4% (338/378)] (Table 1).

### Biologic initiation

Significantly more AS patients [46% (295/641)] were treated with biologics compared with PsA patients [28.8% (378/1312)]. The mean time to biologic treatment from diagnosis was 6.3 years (s.d. 4.8) in AS patients and 6.2 years (s.d. 4.6) in PsA patients (Table 1). Data on biologic agents used for AS and PsA patients are available in Supplementary Figs S1 and S2, available at *Rheumatology Advances in Practice* online.

### Biologic treatment change/failure

The rate of biologic treatment failure, as defined by individuals who stopped, added or switched biologic medication, was 23.1% (68/295) in AS patients and 22.2% (84/378) in PsA patients (Table 1).

### Comorbidities, alcohol use and smoking status

There was no significant difference in comorbidities, use of alcohol and smoking status between AS and PsA patients (Table 1).

### Healthcare use pre- and post-biologic use

#### GP visits

Involvement with the GP (visits, prescriptions, administrative tasks) was significantly reduced from pre- to post-biologic treatment for both AS [difference 60.9 (95% CI 44.2, 77.6)] and PsA [difference 64.4 (95% CI 44.9, 83.9)] (Table 2).

**Table 2:** GP involvement, sickness and disability pre- and post-biologic treatment

Variable	1 year pre-biologic	1 year post-biologic	Difference (95% CI)
GP involvement attended, mean (s.d.)			
AS (*n* = 295)	69.7 (143.5)	8.8 (26.3)	60.9 (44.2, 77.6)^*^
PsA (*n* = 378)	75.1 (167.4)	10.7 (36.3)	64.4 (44.9, 83.9)^*^
Proportion of patients issued sick notes by GP, % (*n*)			
AS (*n* = 295)	5.8 (17)	<1.7	>4.1 (1.0, 7.5)^*^
PsA (*n* = 378)	5.3 (20)	<1.4	>4.0 (1.4, 6.8)^*^
Proportion of patients receiving disability living allowance, % (*n*)			
AS (*n* = 295)	15.9 (47)	<1.7	>14.2 (9.9, 18.9)^*^
PsA (*n* = 378)	15.6 (59)	<1.4	>14.3 (10.6, 18.4)^*^

* *P* < 0.05. ^†^Data suppressed to protect patient anonymity as <5 records.

#### Rheumatology visits

Visits to the rheumatologist remained unchanged pre- and post-biologic for AS and PsA patients [1.0 (s.d. 0.2)] (Table 1)

#### Sick notes

The rate of sick notes issued in primary care within 1 year before commencing biologic therapy for AS and PsA patients was 5.8% (17/295) and 5.3% (20/378), respectively. These figures decreased the 1 year post-biologic start date to less than five individuals (<1.3%) having a sick note (Table 2).

#### Disability payments

Disability-related payments were issued to 15.9% of AS patients and 15.6% of PsA patients within 1 year prior to commencing biologic treatment. This rate decreased to <1.3% 1 year following biologic treatment (Table 2).

### Factors associated with commencing biologic treatment

A Cox proportional hazards model showed two factors that were associated with commencing biologic treatment in AS patients: prior disability-related payments [HR 4.26 (95% CI 3.02, 6.00)] and sick notes [HR 1.60 (95% CI 1.14, 2.24)] (Supplementary Table S5, available at *Rheumatology Advances in Practice* online).

For PsA patients, factors associated with commencing biologic therapy were prior disability-related payments [HR 2.55 (95% CI 1.15, 5.66)] and an incremental increased risk of commencing biologics per each additional DMARD used [HR 2.21 (95% CI 1.87, 2.60)] (Supplementary Table S6, available at *Rheumatology Advances in Practice* online).

### Factors associated with biologic treatment failure

The duration of biologic treatment was the only significant factor associated with both AS and PsA biologic treatment failure in their respective Cox proportional hazards models (see Supplementary Tables S7 and S8, available at *Rheumatology Advances in Practice* online). For AS patients, with each additional year of biologic treatment there was an incremental increased risk of treatment failure of 51% [HR 1.51 (95% CI 1.39, 1.64)] (Supplementary Table S7, available at *Rheumatology Advances in Practice* online). This was also the case for PsA patients, with an increased risk of 32% for each additional year of biologic treatment [HR 1.32 (95% CI 1.24, 1.42)] (Supplementary Table S8, available at *Rheumatology Advances in Practice* online).

## Discussion

Our real-world data found that the time from symptom onset, either peripheral joint or back pain, to diagnosis in patients with PsA and AS is 6–9 years. For AS, this is similar to the findings from the DANBIO registry (88 months) [22], while for PsA the delay is longer (41 months).

The impact of AS and PsA on work disability has been documented previously [8, 10, 11, 13–16, 23]. Sick leave is reportedly high for AS patients, with 63% experiencing at least one period of sick leave and 45% experiencing recurrent sick leave [24]. For PsA, findings from clinical trials and cohort studies estimate unemployment rates between 20 and 50% and work disability rates between 16 and 39% [8]. Registry data on AS patients from the UK have demonstrated that biologics provide greater improvements in presenteeism and work impairment [25]. Our data demonstrate a decreased rate of sick notes issued after biologic treatment.

The impact of work disability and sick leave is huge for society and the individual. For instance, the indirect costs associated with sick leave and work disability due to AS are at least as high as direct costs [26]. In addition to costs associated with the loss of working hours, early retirement and unpaid caregivers’ time are also significant, and increased functional impairment is associated with even greater costs of AS [27]. Predictors of sick leave in AS are disease activity and physical function, however, only in AS patients with lower educational attainment [24].

Here we found that the use of biologics is associated with a reduction in sick notes issued in primary care in both AS and PsA patients. This provides real-world evidence from routinely collected health data confirming the benefit of biologic treatment on work productivity as observed in clinical trials, registry data, meta-analyses and cohort studies [25, 28–30].

In addition, payments issued in the UK to assist with chronic and prolonged disability were reduced after biologics compared with before. This provides evidence that in the real-world setting, biologics are associated with improving disease symptoms and functional ability in severe AS and PsA patients.

Healthcare visits/interactions with the GP deceased significantly within 1 year of commencing biologic treatment compared with the year preceding treatment, suggesting optimal disease control and related reduced healthcare utilization. Although it could be argued that the patient is now seeing his/her secondary care physician more, causing a decrease in GP encounters, the number of appointments in rheumatology clinics post-biologics did not increase.

Biologic medications were more widely used in AS patients compared with PsA patients. The prevalence of biologics use is similar to that observed in other countries [31, 32]. The rate of treatment failure/change in the biologic treatment regimen for all patients was ∼21%, which is the same proportion of patients found to switch to a different biologic in a study conducted in the USA using medical claims data [33]. In accordance with NICE guidelines, AS patients commonly used NSAIDs while PsA patients frequently used DMARDS prior to beginning biologic medication.

The proportion of AS patients with early peripheral joint pain observed in our study was higher than reported elsewhere [34, 35]. However, a recent study has observed high rates of presenting peripheral joint pain in early, ‘pre-diagnosed AS’ [36]. In addition, the proportion of PsA patients with early back pain is also higher than expected in our study. However, there is a lack of high-quality population epidemiology for comparison. A recent MRI study detected sacroiliitis in 38% of patients with PsA, which suggests this is an underrecognized manifestation [37].

Biologic therapy has been shown to lead to short- and long-term improvements in disease activity and health-related quality of life in AS [38–40] and PsA [41–43]. In PsA patients, treatment with biologics has been shown to improve work disability, clinical outcomes and participation in social activities [29, 30, 44].

Factors that were associated with commencing biologics in AS patients were sick notes and disability payments, which are suggestive of poorly controlled disease and impaired function. Similarly for PsA patients, disability payments were also associated with starting biologic treatment, in addition to increased DMARDs used pre-biologics, which are also likely to indicate increased disease activity and impaired function. Biologic treatment failure was associated with increased biologic treatment duration, as one might expect due to increased chance of failure because of loss of effect with sustained biologic treatment [45].

### Strengths

This study uses real-life routinely collected health data. As such, medical events are confirmed in the data with the presence of clinical coding as opposed to relying on participant memory of self-reported events. Using real-life patient data from rheumatology centres in Wales, the findings are also likely to be representative of individuals under the care of a rheumatologist in the UK rather than, for instance, more severe patients who tend to be enrolled in randomized controlled trials.

The findings that biologics reduce the rate of sick notes (improving disease activity), reduces the need for disability-related payments (improving disease activity and functional ability) and reduces visits to the GP (improving disease activity and reducing primary care healthcare utilization) demonstrates their utility to improve patient quality of life and reduce work disability in AS and PsA patients.

### Weaknesses

Treatment failure was not accessible to us from the routine data since end dates of treatments are not recorded. As such, switching medication or adding additional therapy was used as a proxy to define treatment failure. We were unable to discriminate between primary care encounters from the routine data, which would have been useful to assess health resource utilization. We were also not able to assess the dosage of biologic medications, so we could not determine dose escalation.

## Conclusion

It is important to identify and remove barriers for the timely diagnosis of AS and PsA to facilitate earlier diagnosis and treatment to help prevent disability and improve quality of life. However, in a cohort of individuals with a large diagnosis delay, the effect of biologics on outcome, in particular, reduction of long-term sickness as evidenced by decreased sick notes is extremely positive. Interventions aimed at reducing functional impairment can help reduce work disability and the associated costs while improving employment prospects and quality of life for AS and PsA patients.

## Supplementary Material

rkab042_Supplementary_Data
