# Supplementary material for: Biologic use in psoriatic arthritis and ankylosing spondylitis patients: a descriptive epidemiological study using linked, routine data in Wales, UK
Source: Rheumatol Adv Pract. 2021 Jun 27;5(2):rkab042. doi: 10.1093/rap/rkab042 (PMC8496109; doi:10.1093/rap/rkab042)

Supplementary Table S1: Definition of conditions, drugs and events from primary care records using the READ code system

READ_CD	DESCRIPTION (CARDIOVASCULAR DISEASE)
14A3.	H/O: myocardial infarct <60
14A4.	H/O: myocardial infarct >60
14A5.	H/O: angina pectoris
14A6.	H/O: heart failure
14AH.	H/O: Myocardial infarction in last year
14AJ.	H/O: Angina in last year
14AM.	H/O: Heart failure in last year
14AT.	History of myocardial infarction
14AW.	H/O acute coronary syndrome
14NB.	H/O: Peripheral vascular disease procedure
1J60.	Suspected heart failure
1O1..	Heart failure confirmed
21264	Heart failure resolved
323..	ECG: myocardial infarction
3232	ECG: old myocardial infarction
323Z.	ECG: myocardial infarct NOS
388D.	New York Heart Assoc classification heart failure symptoms
661M5	Heart failure self-management plan agreed
662p.	Heart failure 6 month review
662T.	Congestive heart failure monitoring
662W.	Heart failure annual review
679W1	Education about deteriorating heart failure
679X.	Heart failure education
67D4.	Heart failure information given to patient
792..	Coronary artery operations
7920	Saphenous vein graft replacement of coronary artery
7921	Other autograft replacement of coronary artery
7922	Allograft replacement of coronary artery
7923	Prosthetic replacement of coronary artery
7924	Revision of bypass for coronary artery
79240	Revision of bypass for one coronary artery
79241	Revision of bypass for two coronary arteries
79242	Revision of bypass for three coronary arteries
79243	Revision of bypass for four or more coronary arteries
7924y	Other specified revision of bypass for coronary artery
7924z	Revision of bypass for coronary artery NOS
7925	Connection of mammary artery to coronary artery
79275	Open angioplasty of coronary artery
7928	Transluminal balloon angioplasty of coronary artery
79280	Percut transluminal balloon angioplasty one coronary artery
79281	Percut translum balloon angioplasty mult coronary arteries
79282	Percut translum balloon angioplasty bypass graft coronary a

79283	Percut translum cutting balloon angioplasty coronary artery
7928y	Transluminal balloon angioplasty of coronary artery OS
7928z	Transluminal balloon angioplasty of coronary artery NOS
79290	Percutaneous transluminal laser coronary angioplasty
79293	Rotary blade coronary angioplasty
79294	Insertion of coronary artery stent
79295	Insertion of drug-eluting coronary artery stent
792D.	Other bypass of coronary artery
792Dy	Other specified other bypass of coronary artery
792Dz	Other bypass of coronary artery NOS
793G.	Perc translumin balloon angioplasty stenting coronary artery
793Gy	OS perc translumina balloon angioplast stenting coronary art
793Gz	Perc translum balloon angioplasty stenting coronary art NOS
889A.	Diab mellit insulin-glucose infus acute myocardial infarct
8CeC.	Preferred place of care for next exacerbation heart failure
8CL3.	Heart failure care plan discussed with patient
8CMK.	Has heart failure management plan
8CMW8	Heart failure clinical pathway
8H2S.	Admit heart failure emergency
8HBE.	Heart failure follow-up
8HBJ.	Stroke / transient ischaemic attack referral
8L40.	Coronary artery bypass graft operation planned
8L41.	Coronary angioplasty planned
G1yz1	Rheumatic left ventricular failure
G232.	Hypertensive heart&renal dis wth (congestive) heart failure
G3...	Ischaemic heart disease
G30..	Acute myocardial infarction
G301.	Other specified anterior myocardial infarction
G301z	Anterior myocardial infarction NOS
G304.	Posterior myocardial infarction NOS
G305.	Lateral myocardial infarction NOS
G306.	True posterior myocardial infarction
G3071	Acute non-ST segment elevation myocardial infarction
G308.	Inferior myocardial infarction NOS
G30B.	Acute posterolateral myocardial infarction
G30X.	Acute transmural myocardial infarction of unspecif site
G30X0	Acute ST segment elevation myocardial infarction
G30y.	Other acute myocardial infarction
G30yz	Other acute myocardial infarction NOS
G30z.	Acute myocardial infarction NOS
G31..	Other acute and subacute ischaemic heart disease
G310.	Postmyocardial infarction syndrome
G311.	Preinfarction syndrome
G3110	Myocardial infarction aborted
G3111	Unstable angina
G3113	Refractory angina

G3115	Acute coronary syndrome
G31y.	Other acute and subacute ischaemic heart disease
G31y0	Acute coronary insufficiency
G31yz	Other acute and subacute ischaemic heart disease NOS
G32..	Old myocardial infarction
G33..	Angina pectoris
G331.	Prinzmetal's angina
G33z.	Angina pectoris NOS
G33z3	Angina on effort
G33z5	Post infarct angina
G33z7	Stable angina
G33zz	Angina pectoris NOS
G34..	Other chronic ischaemic heart disease
G340.	Coronary atherosclerosis
G34y.	Other specified chronic ischaemic heart disease
G34yz	Other specified chronic ischaemic heart disease NOS
G34z.	Other chronic ischaemic heart disease NOS
G35..	Subsequent myocardial infarction
G350.	Subsequent myocardial infarction of anterior wall
G351.	Subsequent myocardial infarction of inferior wall
G353.	Subsequent myocardial infarction of other sites
G35X.	Subsequent myocardial infarction of unspecified site
G36..	Certain current complication follow acute myocardial infarct
G38..	Postoperative myocardial infarction
G380.	Postoperative transmural myocardial infarction anterior wall
G381.	Postoperative transmural myocardial infarction inferior wall
G383.	Postoperative transmural myocardial infarction unspec site
G384.	Postoperative subendocardial myocardial infarction
G38z.	Postoperative myocardial infarction, unspecified
G3y..	Other specified ischaemic heart disease
G3z..	Ischaemic heart disease NOS
G58..	Heart failure
G580.	Congestive heart failure
G5800	Acute congestive heart failure
G5801	Chronic congestive heart failure
G5804	Congestive heart failure due to valvular disease
G581.	Left ventricular failure
G5810	Acute left ventricular failure
G582.	Acute heart failure
G583.	Heart failure with normal ejection fraction
G584.	Right ventricular failure
G58z.	Heart failure NOS
G5y4z	Post cardiac operation heart failure NOS
G670.	Cerebral atherosclerosis
G677.	Occlusion/stenosis cerebral arts not result cerebral infarct
G70..	Atherosclerosis

G700.	Aortic atherosclerosis
G701.	Renal artery atherosclerosis
G70y0	Carotid artery atherosclerosis
Gyu3.	[X]Ischaemic heart diseases
Gyu30	[X]Other forms of angina pectoris
Gyu32	[X]Other forms of acute ischaemic heart disease
Gyu33	[X]Other forms of chronic ischaemic heart disease
Gyu34	[X]Acute transmural myocardial infarction of unspecif site
Gyu36	[X]Subsequent myocardial infarction of unspecified site
ZV457	[V]Presence of aortocoronary bypass graft
ZV458	[V]Presence of coronary angioplasty implant and graft
ZV45K	[V]Presence of coronary artery bypass graft

READ_CD	DESCRIPTION (DIABETES)
C1001	Diabetes mellitus, adult onset, no mention of complication
C1011	Diabetes mellitus, adult onset, with ketoacidosis
C1021	Diabetes mellitus, adult onset, with hyperosmolar coma
C1031	Diabetes mellitus, adult onset, with ketoacidotic coma
C1041	Diabetes mellitus, adult onset, with renal manifestation
C1051	Diabetes mellitus, adult onset, + ophthalmic manifestation
C1061	Diabetes mellitus, adult onset, + neurological manifestation
C1071	Diabetes mellitus, adult, + peripheral circulatory disorder
C1072	Diabetes mellitus, adult with gangrene
C1074	NIDDM with peripheral circulatory disorder
C109.	Non-insulin dependent diabetes mellitus
C1090	Non-insulin-dependent diabetes mellitus with renal comps
C1091	Non-insulin-dependent diabetes mellitus with ophthalm comps
C1092	Non-insulin-dependent diabetes mellitus with neuro comps
C1093	Non-insulin-dependent diabetes mellitus with multiple comps
C1094	Non-insulin dependent diabetes mellitus with ulcer
C1095	Non-insulin dependent diabetes mellitus with gangrene
C1096	Non-insulin-dependent diabetes mellitus with retinopathy
C1097	Non-insulin dependent diabetes mellitus - poor control
C1099	Non-insulin-dependent diabetes mellitus without complication
C109A	Non-insulin dependent diabetes mellitus with mononeuropathy
C109B	Non-insulin dependent diabetes mellitus with polyneuropathy
C109C	Non-insulin dependent diabetes mellitus with nephropathy
C109D	Non-insulin dependent diabetes mellitus with hypoglyca coma
C109E	Non-insulin depend diabetes mellitus with diabetic cataract
C109F	Non-insulin-dependent d m with peripheral angiopath
C109G	Non-insulin dependent diabetes mellitus with arthropathy
C109H	Non-insulin dependent d m with neuropathic arthropathy
C109J	Insulin treated Type 2 diabetes mellitus
C109K	Hyperosmolar non-ketotic state in type 2 diabetes mellitus
C10F.	Type 2 diabetes mellitus
C10F0	Type 2 diabetes mellitus with renal complications
C10F1	Type 2 diabetes mellitus with ophthalmic complications

C10F2	Type 2 diabetes mellitus with neurological complications
C10F3	Type 2 diabetes mellitus with multiple complications
C10F4	Type 2 diabetes mellitus with ulcer
C10F5	Type 2 diabetes mellitus with gangrene
C10F6	Type 2 diabetes mellitus with retinopathy
C10F7	Type 2 diabetes mellitus - poor control
C10F9	Type 2 diabetes mellitus without complication
C10FA	Type 2 diabetes mellitus with mononeuropathy
C10FB	Type 2 diabetes mellitus with polyneuropathy
C10FC	Type 2 diabetes mellitus with nephropathy
C10FD	Type 2 diabetes mellitus with hypoglycaemic coma
C10FE	Type 2 diabetes mellitus with diabetic cataract
C10FF	Type 2 diabetes mellitus with peripheral angiopathy
C10FG	Type 2 diabetes mellitus with arthropathy
C10FH	Type 2 diabetes mellitus with neuropathic arthropathy
C10FJ	Insulin treated Type 2 diabetes mellitus
C10FK	Hyperosmolar non-ketotic state in type 2 diabetes mellitus
C10FL	Type 2 diabetes mellitus with persistent proteinuria
C10FM	Type 2 diabetes mellitus with persistent microalbuminuria
C10FN	Type 2 diabetes mellitus with ketoacidosis
C10FQ	Type 2 diabetes mellitus with exudative maculopathy
C10FR	Type 2 diabetes mellitus with gastroparesis
C10y1	Diabetes mellitus, adult, + other specified manifestation
C10z1	Diabetes mellitus, adult onset, + unspecified complication
L1806	Pre-existing diabetes mellitus, non-insulin-dependent

READ_CD	DESCRIPTION (DIABETES)
G200.	Malignant essential hypertens.
G220.	Malignant hypertens.renal dis.
G2100	Malig.hypert.heart dis.-no CCF
G210z	Malig.hypertens.heart dis. NOS
G2111	Benign hypert.heart dis-+ CCF
G2400	Second.malig.renovasc.hypert.
G240z	Secondary malign.hypertens.NOS
G201.	Benign essential hypertension
G221.	Benign hypertensive renal dis.
G21z0	Hypertens.heart dis.NOS-no CCF
G24z0	Secondary renovasc.hypert. NOS
G2...	Hypertensive disease
G20..	Essential hypertension
G22..	Hypertensive renal disease
G26..	Severe hypertensin (NICE 2011)
G27..	Hypertnsn resistnt to drg ther
G2y..	Hypertensive disease OS
G20z.	Essential hypertension NOS
G22z.	Hypertensive renal disease NOS
G203.	Diastolic hypertension

G232.	Hypert ht&ren d+(congs)ht fail
G234.	Hyp ht&ren d+both(con)h&r fail
G244.	Hypertens 2ndry endocrin disor
G202.	Systolic hypertension
G21z1	Hypertens.heart dis.NOS- + CCF
G222.	Hypertens renal dis+renal fail
G233.	Hypertn hrt&ren dis+renal fail
G24z1	Hypertension secondary to drug
G21zz	Hypertensive heart disease NOS
G24zz	Secondary hypertension NOS
G21..	Hypertensive heart disease
G2101	Malig.hypert.heart dis.-+ CCF
G2110	Benign hypert.heart dis-no CCF
G211z	Benign hypertens.heart dis.NOS
G23..	Hypertensive heart+renal dis.
G24..	Secondary hypertension
G2410	Second.benign renovasc.hypert.
G241z	Secondary benign hypertens.NOS
G25..	Stge 1 hypertensin (NICE 2011)
G28..	Stge 2 hypertensin (NICE 2011)
G2z..	Hypertensive disease NOS
G211.	Benign hypertensive heart dis.
G231.	Benign hypert.heart+renal dis.
G241.	Secondary benign hypertension
G251.	Stage 1 hyp wi ev end org dmge
G21z.	Hypertensive heart disease NOS
G23z.	Hypertens.heart+renal dis.NOS
G24z.	Secondary hypertension NOS
G210.	Malignant hypertens.heart dis.
G230.	Malig.hypert.heart+renal dis.
G240.	Secondary malignant hypertens.
G250.	Stage 1 hyp wo ev end org dmge

READ_CD	DESCRIPTION (Hyperlipidaemia)
C324.	Hyperlipidaemia NOS
C322.	Mixed hyperlipidaemia
C3201	Hyperbetalipoproteinaemia
C3203	LDL hyperlipoproteinaemia
C320.	Pure hypercholesterolaemia
C320y	Pure hypercholesterolaemia OS
C3200	Familial hypercholesterolaemia
C320z	Pure hypercholesterolaemia NOS
C3202	Hyperlipidaemia, group A
C3206	Polygenic hypercholesterolemia
C3205	Fam defect apolipoprot B-100
C3204	Fredrickson type IIa lipidaem
C328.	Dyslipidaemia

C321.	Pure hyperglyceridaemia
C3210	Hypertriglyceridaemia
READ_CD	DESCRIPTION (INTERSTITIAL LUNG DISEASE)
H58y3	Interstitial lung disease (NEC)
H58y7	Interstitial lung disease due to connective tissue disease
H58y5	Respiratory bronchiolitis associated with Interstitial lung disease
H58y6	Interstitial lung disease due to collagen vascular disease
READ_CD	DESCRIPTION (BACKPAIN)
16C9.	Chronic low back pain
N142.	Pain in lumbar spine
N127.	Lumbar disc degeneration
N12z.	Other/unspec.disc disrders
8HTH.	Referral to back pain clinic
N12C.	Disc prolapse + radiculopathy
N122.	Lumbar disc replacement
N123.	Disc unsp. displ. – no myelopathy
N143.	Sciatica
16C6.	Back pain without radiat NOS
16C7.	Upper backache
N128.	Degenerative disc disease NOS
N12D.	Narrowing disc space
N1420	Lumbago with sciatica
N145.	Backache unspecified
N141.	Pain in thoracic spine
READ_CD	DESCRIPTION (BACKPAIN)
1M10.	Knee pain
N2450	Hand pain
N2451	Foot pain
N2452	Pain in leg
N2454	Calf pain
N2455	Axillary pain
N2456	Tender heel pad
N2457	Shoulder pain
1M13.	Ankle pain
1M01.	Wrist pain
READ_CD	DESCRIPTION (NSAID)
j2m..	Aceclofenac
j2m2.	Aceclofenac 100mg tablet
j2m1.	Preservex 100mg tablet
j2j..	Acemetacin
j2j1.	Acemetacin 60mg capsule
j2j2.	Emflex 60mg capsule
x02LX	Aspirin
x04tL	Oral aspirin
j111.	Aspirin 300mg tablet
di15.	Claradin 300mg tablet

di16.	Laboprin 300mg tablet
di11.	Aspirin [CNS] 300mg tablet
j112.	Aspirin 300mg dispersible tablet
di1r.	Disprin 300mg dispersible tablet
di18.	Solprin 300mg dispersible tablet
di12.	Aspirin [CNS] 300mg dispersible tablet
bu27.	Aspirin 300mg effervescent tablet
bu26.	Platet 300mg effervescent tablet
di1m.	Aspirin 300mg soluble tablet
di1f.	Aspirin 300mg e/c tablet
di1k.	Caprin 300mg e/c tablet
di1c.	Nu-Seals Aspirin 300mg e/c tablet
bu2b.	Aspirin 300mg m/r tablet
bu2a.	Disprin CV 300mg m/r tablet
di1h.	Aspirin 324mg e/c tablet
di1a.	Caprin 324mg e/c tablet
di19.	Aspirin 500mg m/r tablet
x02AA	Anadin All-Night 500mg m/r tablet
di1b.	Levius 500mg m/r tablet
di1p.	Aspirin 500mg soluble tablet
di1q.	Aspro Clear Maximum Strength soluble tablet
di1i.	Aspirin 600mg tablet
di17.	Paynocil 600mg tablet
di1e.	Palaprin Forte 600mg tablet
di1g.	Aspirin 600mg e/c tablet
di1d.	Nu-Seals Aspirin 600mg e/c tablet
bu2I.	Aspirin 162.5mg m/r capsule
bu2J.	Caspac XL 162.5mg m/r capsule
di14.	Aspergum 227mg chewing gum
di1j.	Laboprin DL 900mg sachet
x01Kv	Aspirin tablets
x01Kw	Rectal Aspirin
di1o.	Aspirin 150mg suppository
di1n.	Aspirin 300mg suppository
x01Kx	Aspirin compound preparation
x000x	Co-codaprin
dia4.	Co-codaprin 8mg/400mg tablet
dia5.	Co-codaprin 8mg/400mg dispersible tablet
diab.	Codis dispersible tablet
diaO.	Co-codaprin 8mg/500mg dispersible tablet
diaP.	Codis 500 dispersible tablet
x00et	Aspirin+codeine 300mg/8mg tablet
x00l7	Disprin Direct dispersible tablet
dibA.	Aspirin+methocarbamol 325mg/400mg tablet
diaG.	Aspirin+papaveretum 500mg/7.71mg dispersible tablet
dia9.	Aspav dispersible tablet

diay.	Anadin tablet
diaD.	Anadin Extra tablet
dia8.	Antoin dispersible tablet
diaz.	Askit powder
diae.	Doloxene Compound capsule
diaf.	Equagesic tablet
diai.	Hypon tablet
dicH.	Phensic tablet
dibq.	Trancoprin tablet
dia1.	Aspirin/paracetamol/codeine tablets
diat.	Aspirin+papaveretum 500mg/10mg dispersible tablet
di1..	Aspirin [central nervous system use]
j11..	Aspirin [musculoskeletal use]
x02Mm	Benorilate
j12x.	Benorilate 750mg tablet
j121.	Benoral 750mg tablet
j12y.	Benorilate 2g/sachet granules
j122.	Benoral 2g/sachet granules
j12z.	Benorilate 2g/5mL s/f suspension
x00eL	Benoral 2g/5mL s/f suspension
j123.	Benoral 2g/5mL sugar free suspension 150mL
j124.	Benoral 2g/5mL sugar free suspension 300mL
di3..	Benorylate [central nervous sytem use]
j12..	Benorilate [musculoskeletal use]
jA2..	Get from Read code browser/ref
j2t..	Dexibuprofen
j2ty.	Dexibuprofen 300mg tablet
j2t2.	Seractil 300mg tablet
j2tz.	Dexibuprofen 400mg tablet
j2t1.	Seractil 400mg tablet
j2q..	Dexketoprofen
j2qz.	Dexketoprofen 25mg tablet
j2q1.	Keral 25mg tablet
x01Ky	Diclofenac
x01Kz	Oral diclofenac
j2ry.	Diclofenac potassium 25mg tablet
j2r1.	Voltarol Rapid 25mg tablet
j2rz.	Diclofenac potassium 50mg tablet
j2r2.	Voltarol Rapid 50mg tablet
j22d.	Diclofenac sodium 25mg e/c tablet
j2oC.	Acoflam 25mg e/c tablet
j2oo.	Defanac 25mg e/c tablet
j22I.	Dicloflex 25mg e/c tablet
j2o4.	Diclovol 25mg e/c tablet
j22l.	Diclozip-25 e/c tablet
j2oq.	Fenactol 25mg e/c tablet

j22w.	Flamrase 25mg e/c tablet
j22L.	Lofensaid 25 tablet
j227.	Rhumalgan 25mg e/c tablet
j22j.	Valenac 25mg e/c tablet
j22f.	Volraman 25mg e/c tablet
x00LD	Voltarene 25mg e/c tablet
j221.	Voltarol 25mg e/c tablet
j22u.	Diclofenac sodium 25mg tablets
j22e.	Diclofenac sodium 50mg e/c tablet
j2oB.	Acoflam 50mg e/c tablet
j2op.	Defanac 50mg e/c tablet
j22J.	Dicloflex 50mg e/c tablet
j2o5.	Diclovol 50mg e/c tablet
j22m.	Diclozip-50 e/c tablet
j2or.	Fenactol 50mg e/c tablet
j22x.	Flamrase 50mg e/c tablet
j22s.	Isclofen 50mg e/c tablet
j22M.	Lofensaid 50 tablet
j228.	Rhumalgan 50mg e/c tablet
j22k.	Valenac 50mg e/c tablet
j22g.	Volraman 50mg e/c tablet
x00LE	Voltarene 50mg e/c tablet
j222.	Voltarol 50mg e/c tablet
j22v.	Diclofenac sodium 50mg tablets
j22i.	Diclofenac sodium 50mg dispersible tablet
j22h.	Voltarol 50mg dispersible tablet
j22y.	Diclofenac sodium 75mg m/r tablet
j2oA.	Acoflam SR 75mg m/r tablet
j2ol.	Defanac SR 75mg m/r tablet
j2o8.	Dexomon SR 75mg m/r tablet
j2o1.	Dicloflex SR 75mg m/r tablet
j2o6.	Diclovol SR 75mg m/r tablet
j22Y.	Diclotard 75 m/r tablet
j2ow.	Econac SR 75mg tablet
j2os.	Fenactol SR 75mg m/r tablet
j2o2.	Flamatak MR 75mg m/r tablet
j2oG.	Flamrase SR 75mg m/r tablet
j2oF.	Rheumatac Retard 75mg m/r tablet
j22O.	Rhumalgan CR 75mg m/r tablet
j22S.	Lofensaid Retard 75 m/r tablet
j22V.	Slofenac SR 75mg m/r tablet
j2ou.	Valdic 75 Retard 75mg m/r tablet
j22P.	Volsaid Retard 75mg m/r tablet
j22n.	Voltarol SR 75mg m/r tablet
j22C.	Diclofenac sodium 75mg m/r capsule
j22H.	Diclomax SR 75mg m/r capsule

j2oM.	Rhumalgan SR 75mg m/r capsule
j22R.	Diclofenac sodium 75mg e/c+m/r capsule
j22B.	Motifene 75mg e/c+m/r capsule
j22c.	Diclofenac sodium 100mg m/r tablet
j2oD.	Acoflam Retard 100mg m/r tablet
j2oJ.	Closteril 100 m/r tablet
j2om.	Defanac Retard 100mg m/r tablet
j2o9.	Dexomon Retard 100mg m/r tablet
j22K.	Dicloflex Retard 100mg m/r tablet
j22Z.	Diclotard 100 m/r tablet
j2o7.	Diclovol Retard 100mg m/r tablet
j2o3.	Difenor XL 100mg m/r tablet
j22W.	Digenac XL 100 m/r tablet
j2ox.	Econac XL 100mg tablet
j2ot.	Fenactol Retard 100mg m/r tablet
j2oE.	Flamatak MR 100mg m/r tablet
j22A.	Flamrase SR 100mg m/r tablet
j22X.	Flexotard MR 100mg m/r tablet
j22T.	Lofensaid Retard 100 m/r tablet
j22N.	Rhumalgan CR 100mg m/r tablet
j22U.	Slofenac SR 100mg m/r tablet
j2ov.	Valdic 100 Retard 100mg m/r tablet
j22Q.	Volsaid Retard 100mg m/r tablet
x00Ts	Voltaren Retard 100mg m/r tablet
x00Tt	Voltarene LP 100mg m/r tablet
j226.	Voltarol Retard 100mg m/r tablet
j22r.	Diclofenac sodium 100mg m/r capsule
j22q.	Diclomax Retard 100mg m/r capsule
j2oL.	Rhumalgan XL 100mg m/r capsule
x01L0	Rectal diclofenac
j22a.	Diclofenac sodium 100mg suppository
j2oH.	Econac 100mg suppository
j224.	Voltarol 100mg suppository
j22F.	Diclofenac sodium 25mg suppository
j22D.	Voltarol 25mg suppository
j22G.	Diclofenac sodium 50mg suppository
j22E.	Voltarol 50mg suppository
j22b.	Diclofenac sodium 12.5mg paediatric suppository
j225.	Voltarol 12.5mg paediatric suppository
x01L1	Parenteral diclofenac
j229.	Diclofenac sodium 75mg/3mL injection
j2oK.	Econac 75mg/3mL injection
j223.	Voltarol 75mg/3mL injection
j2oO.	DICLOFENAC 75mg/2mL solution for injection
j2oN.	DYLOJECT 75mg/2mL solution for injection
x00xb	Topical diclofenac

ja1Y.	Diclofenac sodium 1% gel
x00C5	Voltarol Emulgel
ja1A.	Voltarol Emulgel Topical gel 100g
ja1F.	Voltarol Emulgel Topical gel 20g
x05d3	Voltarol Emulgel P topical gel
ja2g.	Voltarol Emulgel P topical gel 30g
ja2m.	Voltarol Emulgel P topical gel 50g
x05wX	Voltarol Pain-eze Emulgel 1% topical gel
ja2q.	Voltarol Pain-eze Emulgel 1% topical gel 30g
ja2w.	Diclofenac 1% 10cmx14cm patch
ja2s.	Voltarol Gel 1% 10cmx14cm patch
mt1z.	Diclofenac sodium 3% gel
x05J3	Solaraze 3% gel
mt11.	Solaraze 3% gel 25g
ja2y.	Diclofenac sodium 1.5% topical solution
x05FX	Pennsaid 16mg/mL cutaneous solution
ja2e.	Pennsaid 1.5% topical solution 60mL
k6d5.	Diclofenac sodium 0.1% single-use eye drops
x00ca	Voltarol Ophtha 0.1% single-use eye drops
k6d1.	Voltarol Ophtha 0.1% single-use eye drops x4
k6d2.	Voltarol Ophtha 0.1% single-use eye drops x40
k6d6.	Voltarol Ophtha 0.1% single-use eye drops x5
k6d3.	Diclofenac 0.1% single-use eye drops x4
k6d4.	Diclofenac 0.1% single-use eye drops x40
k6d7.	Diclofenac sodium 0.1% multidose eye drops
x05lh	Voltarol Ophtha 0.1% multidose eye drops
k6d8.	Voltarol Ophtha 0.1% multidose eye drops 5mL
j22..	Diclofenac sodium product
j2o..	Diclofenac sodium 2
j2oP.	VOLTAROL ACTIVE 4% spray 15mL
j2oQ.	VOLTAROL ACTIVE 4% spray 30mL
j2oR.	ENSTAR XL 100mg m/r tablets
j2oy.	MOBIGEL 4% spray 25g
j2oz.	DICLOFENAC SODIUM 4% spray
j2r..	Diclofenac potassium
j2r3.	VOLTAROL PAIN-EZE 12.5mg tablets
j2rx.	DICLOFENAC POTASSIUM 12.5mg tablets
k6d..	Diclofenac sodium [eye]
mt1..	Diclofenac sodium [actinic keratosis]
mt12.	SOLARAZE 3% gel 50g
mt13.	SOLARAZE 3% gel 100g
x02MM	Diflunisal
j233.	Diflunisal 250mg tablet
j231.	Dolobid 250mg tablet
x00fB	Dolobis 250mg tablet
j234.	Diflunisal 500mg tablet

j232.	Dolobid 500mg tablet
di4..	Diflunisal [CNS analgesic] [see j23..]
j23..	Diflunisal [musculoskeletal use]
j24..	Etodolac
j246.	Etodolac 200mg tablet
j245.	Lodine 200mg tablet
j242.	Ramodar 200mg tablet
j243.	Etodolac 200mg capsule
j24A.	Ebretin 200mg capsule
j241.	Lodine 200mg capsule
j247.	Etodolac 300mg capsule
j24B.	Ebretin 300mg capsule
j24C.	Eccoxolac 300mg capsule
j244.	Lodine 300mg capsule
j249.	Etodolac 600mg m/r tablet
j24D.	ETOPAN XL 600mg m/r tablets
j248.	Lodine SR 600mg m/r tablet
j25..	Fenbufen
j258.	Fenbufen 300mg tablet
j25A.	Fenbuzip 300mg tablet
j253.	Lederfen 300mg tablet
j254.	Lederfen CP 300mg tablets
j257.	Fenbufen 300mg capsule
x00Dx	Cinopal 300mg capsule
j259.	Fenbuzip 300mg capsule
x026C	Lederfen 300mg capsule
j251.	Lederfen 300mg capsules x21
j252.	Lederfen CP 300mg capsules x84
j25y.	Fenbufen 450mg tablet
j25B.	Fenbuzip 450mg tablet
j255.	Lederfen 450mg tablet
j25z.	Fenbufen 450mg effervescent tablet
x026D	Lederfen F 450mg effervescent tablet
j256.	Lederfen F 450mg effervescent tablets 56
x01L2	Felbinac
ja1u.	Felbinac 3% gel
x026W	Traxam gel
ja1S.	Traxam 3% topical gel 100g
ja1v.	Traxam 3% topical gel 50g
x03hC	Traxam Pain Relief gel
ja2K.	Traxam Pain Relief gel 30g
ja1T.	Felbinac 3.17% foam
x026G	Traxam foam
ja1K.	TRAXAM 3.17% foam 100g
x02MN	Fenoprofen
di5z.	Fenoprofen 200mg tablet

di51.	Progesic 200mg tablet
j263.	Fenoprofen 300mg tablet
j261.	Fenopron 300mg tablet
j264.	Fenoprofen 600mg tablet
j262.	Fenopron 600mg tablet
x00fF	Fepron 600mg tablet
di5..	Fenoprofen [analgesic]
j26..	Fenoprofen [musculoskeletal use]
j27..	Flurbiprofen
j27x.	Flurbiprofen 50mg tablet
j271.	Froben 50mg tablet
j27y.	Flurbiprofen 100mg tablet
j272.	Froben 100mg tablet
j275.	Flurbiprofen 200mg m/r capsule
j274.	Froben SR 200mg m/r capsule
lf3z.	Flurbiprofen 8.75mg lozenge
lf33.	Strefen Honey and Lemon 8.75mg lozenge
lf31.	Strefen 8.75mg lozenge
lf32.	Streflam 8.75mg lozenge
j27z.	Flurbiprofen 100mg suppository
j273.	Froben 100mg suppository
x026y	Flurbiprofen sodium 0.03% single-use eye drops
x026x	Ocufen 0.03% single-use eye drops
k6c1.	Ocufen 0.03% single-use eye drops 0.4mL
k6cz.	Flurbiprofen sodium 0.03% single-use eye drops 0.4mL
k6c..	Flurbiprofen sodium [eye]
lf3..	Flurbiprofen [oropharyngeal]
x02MO	Ibuprofen product
x01L3	Oral ibuprofen
j281.	Ibuprofen 200mg tablet
j28W.	Advil 200mg tablet
j28Z.	Anadin Ibuprofen 200mg tablet
j284.	Apsifen 200mg tablet
j28q.	Arthrofen 200 tablet
j287.	Brufen 200mg tablet
j28C.	Cuprofen 200mg tablet
j28b.	Ebufac 200mg tablet
j2pQ.	Galprofen Ibuprofen 200mg Caplet
x00fN	Ibrufhalal 200mg tablet
j2pZ.	IBUCALM 200mg tablets
j28e.	Ibular 200mg tablet
j28g.	Ibumetin 200mg tablet
j28R.	Inoven 200mg Caplet
j2p5.	Librofem 200mg tablet
j28E.	Lidifen 200mg tablet
x00fZ	Migrafen 200mg tablet

j28j.	Motrin 200mg tablet
j2pH.	Nurofen 200mg tablet
j2p1.	Nurofen Advance 200mg tablet
j2pI.	Nurofen 200mg Caplet
j2pD.	Nurofen Mobile 200mg tablet
j2pS.	Nurofen Tension Headache 200mg tablet
x00fi	Pacifene 200mg tablet
j28m.	Paxofen 200mg tablet
x00fn	Phor Pain 200mg tablet
x00fs	Proflex 200mg tablet
j28S.	Relcofen 200mg tablet
j28M.	Rimafen 200mg tablet
j282.	Ibuprofen 400mg tablet
j28X.	Advil Extra Strength 400mg tablet
j285.	Apsifen 400mg tablet
j28r.	Arthrofen 400 tablet
j288.	Brufen 400mg tablet
j28Q.	Cuprofen Maximum Strength 400mg tablet
j28c.	Ebufac 400mg tablet
j2pa.	IBUCALM 400mg tablets
j28K.	Ibumed 400mg tablet
j28h.	Ibumetin 400mg tablet
j28f.	Ibular 400mg tablet
j28D.	Isisfen 400mg tablet
j28F.	Lidifen 400mg tablet
j2pB.	Mandafen 400mg tablet
j28k.	Motrin 400mg tablet
j2pU.	Nurofen Maximum Strength Migraine Pain 400mg Caplet
x00fh	Pacifene 400mg tablet
j28n.	Paxofen 400mg tablet
x00fm	Phor Pain 400mg tablet
j28N.	Rimafen 400mg tablet
j28T.	Relcofen 400mg tablet
j283.	Ibuprofen 600mg tablet
j28L.	Apsifen F 600mg tablet
j286.	Apsifen F 600mg effervescent tablets
j28t.	Arthrofen 600 tablet
j289.	Brufen 600mg tablet
j28i.	Ibumetin 600mg tablet
j28G.	Lidifen 600mg tablet
j2p7.	Mandafen 600mg tablet
j28l.	Motrin 600mg tablet
j28o.	Paxofen 600mg tablet
j28u.	Ibuprofen 800mg tablet
j28p.	Motrin 800mg tablet
j2pJ.	Ibuprofen 200mg capsule

diaW.	Anadin Ultra 200mg capsule
j2pK.	Nurofen 200mg liquid capsule
j28x.	Ibuprofen 200mg m/r capsule
j28y.	Ibuprofen 300mg m/r capsule
j28d.	Fenbid 300mg Spansule
j2p3.	Nurofen Back Pain SR 300mg m/r capsule
x00Tg	Proflex 300mg m/r capsule
j2pW.	IBUPROFEN 400mg capsules
j2pV.	NUROFEN EXTRA STRENGTH 400mg liquid capsules
j2pY.	GALPHARM MAXIMUM STRENGTH IBUPROFEN 400mg liquid capsules
j28B.	Ibuprofen 800mg m/r tablet
j28A.	Brufen Retard 800mg m/r tablet
j28w.	Ibuprofen 100mg/5mL oral suspension
j28a.	Brufen 100mg/5mL syrup
j28Y.	Ibuprofen 100mg/5mL s/f oral suspension
j2pN.	Orbifen 100mg/5mL s/f oral suspension sachets
j28P.	Ibuprofen 600mg/sachet effervescent granules
j28v.	Brufen 600mg/sachet effervescent granules
x006P	Ibuprofen+codeine phosphate
j28V.	Ibuprofen+codeine phosphate 200mg/12.8mg tablet
j2pM.	Cuprofen PLUS tablet
j2pT.	Solpadeine Migraine Ibuprofen & Codeine tablet
j28U.	Solpaflex tablet
j28I.	Nurofen Plus tablet
j28z.	Ibuprofen+codeine phosphate 300mg/20mg m/r tablet
j28H.	Codafen Continus m/r tablet
j28O.	Ibuprofen+codeine phosphate 200mg/12.5mg tablet
x05YT	Oral ibuprofen compound product
j2pX.	ANADIN ULTRA DOUBLE STRENGTH 400mg liquid capsules
x01L4	Topical ibuprofen
ja1x.	Ibuprofen 5% cream
x00HG	Proflex cream
ja1J.	Proflex 5% cream 50g
ja1w.	Proflex 5% cream 100g
x01L5	Proflex Pain Relief cream
ja1R.	Proflex pain relief 5% cream 25g
ja22.	Proflex pain relief 5% cream 30g
ja2U.	Ibuprofen 5% foam
x03wA	Ibuleve 5% mousse
ja2V.	Ibuleve 5% mousse 125g
ja2a.	Ibuleve 5% mousse 75g
x04vt	Ibumousse 5% foam
ja2W.	Ibumousse 5% foam 125g
ja1U.	Ibuprofen 5% gel
x049k	Cuprofen 5% gel
ja2Q.	Cuprofen 5% gel 30g

ja2R.	Cuprofen 5% gel 50g
x03dl	Fenbid 5% gel
ja2H.	Fenbid 5% gel 100g
ja2M.	Fenbid 5% gel 30g
ja2N.	Fenbid 5% gel 50g
x026Q	Ibugel gel
ja1I.	Ibugel gel 100g
ja1W.	Ibuleve gel
x00Ms	Ibuleve Sports gel
x04yw	Nurofen gel
ja2Z.	Nurofen gel 35g
x05q2	Phorpain 5% gel
ja2l.	Phorpain 5% gel 100g
ja2z.	Ibuprofen 10% gel
x05vE	Care Ibuprofen 10% gel
ja2o.	Care Ibuprofen 10% gel 50g
x04xv	Fenbid Forte 10% gel
ja2X.	Fenbid Forte 10% gel 100g
x0588	Ibugel Forte 10% gel
ja2b.	Ibugel Forte 10% gel 100g
x05Bt	Ibuleve Maximum Strength 10% gel
ja2d.	Ibuleve Maximum Strength 10% gel 30g
ja2h.	Ibuleve Maximum Strength 10% gel 50g
x05wW	Lloyds Pharmacy Maximum Strength Ibuprofen 10% gel
ja2p.	Lloyds Pharmacy Maximum Strength Ibuprofen 10% gel 30g
x05Mg	Nurofen Maximum Strength 10% gel
ja2f.	Nurofen Maximum Strength 10% gel 30g
x05gD	Phorpain Gel Maximum Strength 10% gel
ja2i.	Phorpain Gel Maximum Strength 10% gel 30g
x02Dr	Ibuprofen+menthol 5%/3% gel
x02Ds	Deep Relief Ibuprofen gel
ja2A.	Deep Relief Ibuprofen gel 100g
ja2F.	Deep Relief Ibuprofen gel 15g
ja2G.	Deep Relief Ibuprofen gel 50g
ja24.	Ibuprofen 5% spray
ja25.	Ibuleve 5% topical spray
ja23.	Ibuspray topical spray
di6..	Ibuprofen [cns analgesic] [see j28..]
j28..	Ibuprofen [musculoskeletal use]
j2p..	Ibuprofen [musculoskeletal use 2]
j2pb.	NUROFEN EXPRESS SOLUBLE 400mg/sachet oral powder
j2pc.	IBUPROFEN 400mg/sachet oral powder
j29..	Indometacin product
j291.	Indometacin 25mg capsule
j294.	Artracin 25mg capsule
j296.	Imbrilon 25mg capsule

j299.	Indocid 25mg capsule
j29e.	Indoflex 25mg capsule
j29f.	Indolar 25mg capsule
j29r.	Indomax 25 capsule
j29l.	Mobilan 25mg capsule
j29F.	Rimacid 25mg capsule
j292.	Indometacin 50mg capsule
j295.	Artracin 50mg capsule
j297.	Imbrilon 50mg capsule
j29a.	Indocid 50mg capsule
j29g.	Indolar 50mg capsule
j29m.	Mobilan 50mg capsule
j29E.	Indometacin 25mg m/r tablet
j29B.	Flexin-25 Continus m/r tablet
j29z.	Indometacin 25mg m/r capsule
j29j.	Indomod 25mg m/r capsule
j29D.	Indometacin 50mg m/r tablet
j29C.	Flexin-50 Continus m/r tablet
j29A.	Indometacin 75mg m/r tablet
j29p.	Flexin-75 Continus m/r tablet
j29y.	Indometacin 75mg m/r capsule
j29G.	Artracin SR 75mg m/r capsule
j29u.	Berlind 75 Retard 75mg m/r capsule
j29d.	Indocid R 75mg m/r capsule
j29i.	Indolar SR 75mg m/r capsule
j29s.	Indomax 75 SR m/r capsule
j29k.	Indomod 75mg m/r capsule
j29J.	Indotard MR 75mg m/r capsule
j29H.	Maximet SR 75mg m/r capsule
j29K.	Pardelprin MR 75mg m/r capsule
j29t.	Rheumacin LA 75mg m/r capsule
j29o.	Slo-Indo 75mg m/r capsule
j29w.	Indomethacin 25mg/5mL s/f suspension
j29b.	Indocid 25mg/5mL s/f suspension
j293.	Indometacin 100mg suppository
j298.	Imbrilon 100mg suppository
j29c.	Indocid 100mg suppository
j29h.	Indolar 100mg suppository
j29v.	Indometacin 1mg injection (pdr for recon)
j29q.	Indocid PDA 1mg injection (pdr for recon)
j2a..	Ketoprofen
j2a1.	Ketoprofen 50mg capsule
j2a4.	Alrheumat 50mg capsule
j2aE.	Ketonal 50mg capsule
j2a5.	Orudis 50mg capsule
j2ab.	Tiloket 50mg capsule

j2a2.	Ketoprofen 100mg capsule
j2aF.	Ketonal 100mg capsule
j2a6.	Orudis 100mg capsule
j2ay.	Ketoprofen 100mg m/r capsule
j2aQ.	Tiloket CR 100mg m/r capsule
j2aA.	Ketoprofen CR 100mg m/r capsule
j2aC.	Ketovail 100mg m/r capsule
j2aO.	Ketpron XL 100mg m/r capsule
j2a8.	Oruvail 100 m/r capsule
j2aw.	Ketoprofen 150mg m/r capsule
j2aG.	Oruvail 150 m/r capsule
j2az.	Ketoprofen 200mg m/r capsule
j2aJ.	Fenoket 200mg m/r capsule
j2aM.	Jomethid XL 200mg m/r capsule
j2aR.	Tiloket CR 200mg m/r capsule
j2aI.	Ketocid 200 m/r capsule
j2aB.	Ketoprofen CR 200mg m/r capsule
j2aL.	Ketotard 200 XL m/r capsule
j2aD.	Ketovail 200mg m/r capsule
j2aK.	Ketozip CR 200mg m/r capsule
j2aP.	Ketpron XL 200mg m/r capsule
j2aH.	Larafen CR 200mg m/r capsule
j2a9.	Oruvail 200 m/r capsule
j2a3.	Ketoprofen 100mg suppository
j2a7.	Orudis 100mg suppository
j2ax.	Ketoprofen 100mg/2mL injection
j2aa.	Oruvail IM 100mg/2mL injection
ja1V.	Ketoprofen 2.5% gel
x04yU	Tiloket 2.5% gel
ja2Y.	Tiloket 2.5% gel 100g
ja2r.	Tiloket 2.5% gel 50g
x00N4	Oruvail gel
ja1H.	Oruvail gel 100g
ja1O.	Oruvail gel 30g
ja26.	Powergel 2.5% gel
ja28.	Powergel 2.5% gel 50g
ja29.	Powergel 2.5% gel 100g
ja2J.	Powergel 2.5% gel 50gx2
ja31.	Powergel 2.5% dispenser gel 50g
ja32.	Powergel 2.5% dispenser gel 100g
x03hs	Solpaflex gel
ja2L.	Solpaflex gel 30g
j2aS.	VALKET 200 RETARD 200mg m/r capsules
j2ac.	AXORID 100mg/20mg m/r capsules
j2ad.	AXORID 200mg/20mg m/r capsules
j2au.	KETOPROFEN+OMEPRAZOLE 200mg/20mg m/r capsules

j2av.	KETOPROFEN+OMEPRAZOLE 100mg/20mg m/r capsules
x01L6	Ketorolac
x03ec	Oral ketorolac
o4a3.	Ketorolac trometamol 10mg tablet
o4a4.	Toradol 10mg tablet
x03ed	Parenteral ketorolac
o4a5.	Ketorolac trometamol 10mg/1mL injection
o4a6.	Toradol 10mg/1mL injection
o4a1.	Ketorolac trometamol 30mg/1mL injection
o4a2.	Toradol 30mg/1mL injection
x03eb	Topical ketorolac
k6g1.	Ketorolac trometamol 0.5% ophthalmic solution
x03ea	Acular 0.5% ophthalmic solution
k6g2.	Acular 0.5% ophthalmic solution 10mL
k6g..	Ketorolac trometamol [eye]
o4a..	Ketorolac trometamol
j2s..	Lornoxicam
j2sx.	Lornoxicam 8.6mg injection (pdr for recon)
j2s3.	Xefo 8.6mg injection (pdr for recon)
j2sz.	Lornoxicam 4mg tablet
j2s1.	Xefo 4mg tablet
j2sy.	Lornoxicam 8mg tablet
j2s2.	Xefo 8mg tablet
x02MP	Mefenamic acid
j2b1.	Mefenamic acid 250mg capsule
j2b8.	Contraflam 250mg capsule
j2b6.	Dysman-250 capsule
j2b9.	Meflam 250 capsule
j2bB.	Opustan 250mg capsule
j2b2.	Ponstan 250mg capsule
j2bz.	Mefenamic acid 250mg dispersible tablet
j2b5.	Ponstan Dispersible 250mg tablet
j2bx.	Mefenamic acid 500mg tablet
j2bD.	Contraflam 500mg tablet
j2b7.	Dysman-500 tablet
j2bA.	Meflam 500 tablet
j2bC.	Opustan 500mg tablet
j2b3.	Ponstan Forte 500mg tablet
j2by.	Mefenamic acid 50mg/5mL suspension
j2b4.	Ponstan 50mg/5mL paediatric suspension
di7..	Mefenamic acid [cns analgesic] [see j2b..]
j2b..	Mefenamic acid [musculoskeletal use]
j2k..	Nabumetone
j2k2.	Nabumetone 500mg tablet
j2k1.	Relifex 500mg tablet
j2k6.	Nabumetone 500mg dispersible tablet

j2k5.	Relifex 500mg dispersible tablet
j2k4.	Nabumetone 500mg/5mL suspension
j2k3.	Relifex 500mg/5mL suspension
j2c..	Naproxen
j2c1.	Naproxen 250mg tablet
j2cc.	Arthrosin 250 tablet
j2ce.	Arthroxen 250mg tablet
j2c3.	Laraflex 250mg tablet
j2c5.	Naprosyn 250mg tablet
j2cm.	Prosaid 250mg tablet
j2cA.	Rheuflex-250 tablet
j2cH.	Rimoxyn 250mg tablet
j2cI.	Timpron 250mg tablet
j2ca.	Valrox 250mg tablet
j2ch.	Naproxen 375mg tablet
j2cg.	Naprosyn 375mg tablet
j2c2.	Naproxen 500mg tablet
j2cd.	Arthrosin 500 tablet
j2cf.	Arthroxen 500mg tablet
j2c4.	Laraflex 500mg tablet
j2c6.	Naprosyn 500mg tablet
j2cn.	Prosaid 500mg tablet
j2cB.	Rheuflex-500 tablet
j2cG.	Rimoxyn 500mg tablet
j2cF.	Timpron 500mg tablet
j2cb.	Valrox 500mg tablet
j2ck.	Naproxen 250mg e/c tablet
j2cM.	Arthrosin 250mg e/c tablet
j2co.	Naprosyn EC 250mg e/c tablet
j2ci.	Nycopren 250mg e/c tablet
j2cJ.	Timpron 250 EC e/c tablet
j2cw.	Naproxen 375mg e/c tablet
j2cp.	Naprosyn EC 375mg e/c tablet
j2cl.	Naproxen 500mg e/c tablet
j2cN.	Arthrosin 500mg e/c tablet
j2cq.	Naprosyn EC 500mg e/c tablet
j2cj.	Nycopren 500mg e/c tablet
j2cK.	Timpron 500 EC e/c tablet
j2cs.	Naproxen 375mg m/r tablet
j2cC.	Pranoxen Continus 375mg m/r tablet
j2ct.	Naproxen 500mg m/r tablet
j2cE.	Naprosyn SR 500mg m/r tablet
j2cD.	Pranoxen Continus 500mg m/r tablet
di8z.	Naproxen sodium 275mg tablet
di81.	Synflex 275mg tablet
j2cv.	Naproxen 500mg/sachet granules

j2c9.	Naprosyn 500mg/sachet granules
j2cy.	Naproxen 125mg/5mL suspension
j2c7.	Naprosyn 125mg/5mL suspension
j2cz.	Naproxen 500mg suppository
j2c8.	Naprosyn 500mg suppository
di8..	Naproxen sodium
j2cP.	VIMOVO 500mg/20mg m/r tablets
j2cZ.	NAPROXEN+ESOMEPRAZOLE 500mg/20mg m/r tablets
x01L7	Nefopam
di9y.	Nefopam hydrochloride 30mg tablet
di91.	Acupan 30mg tablet
di9z.	Nefopam hydrochloride 20mg/1mL injection
di92.	Acupan 20mg/1mL injection
di9..	Nefopam hydrochloride
j2d..	Phenylbutazone product
j2d1.	Phenylbutazone 100mg tablet
j2d5.	Butazolidin 100mg tablet
j2d7.	Butazone 100mg tablet
x00LC	Phenylbutazone 100mg e/c tablet
j2d3.	Butacote 100mg e/c tablet
j2d2.	Phenylbutazone 200mg tablet
j2d4.	Butacote 200mg tablet
j2d6.	Butazolidin 200mg tablet
j2d8.	Butazone 200mg tablet
j2e..	Piroxicam
j2e1.	Piroxicam 10mg capsule
j2e3.	Feldene 10mg capsule
j2ee.	Flamatrol 10mg capsule
j2e8.	Larapam 10mg capsule
j2eA.	Piroflam 10 capsule
j2ea.	Pirozip 10 capsule
j2e2.	Piroxicam 20mg capsule
j2e4.	Feldene 20mg capsule
j2ef.	Flamatrol 20mg capsule
j2e9.	Larapam 20mg capsule
j2eB.	Piroflam 20 capsule
j2eb.	Pirozip 20 capsule
j2ex.	Piroxicam 10mg dispersible tablet
j2e5.	Feldene 10mg dispersible tablet
j2ey.	Piroxicam 20mg dispersible tablet
j2e6.	Feldene 20mg dispersible tablet
j2ew.	Piroxicam 20mg melt tablet
j2ec.	Feldene Melt 20mg tablet
j2ev.	Piroxicam 20mg/1mL injection
j2ed.	Feldene IM 20mg/1mL injection
j2ez.	Piroxicam 20mg suppository

j2e7.	Feldene 20mg suppository
ja1y.	Piroxicam 0.5% gel
x00Ml	Feldene 0.5% gel
ja1M.	Feldene 0.5% topical gel 112g
ja1z.	Feldene 0.5% topical gel 60g
x03kk	Feldene P 0.5% gel
ja2O.	Feldene P 0.5% gel 30g
ja2P.	Feldene P 0.5% gel 7.5g
x026F	Feldene Sports 0.5% gel
ja1B.	Feldene sports gel 30g
j2i..	Piroxicam-betadex
j2i1.	Piroxicam 20mg tablet
j2i2.	Brexidol 20mg tablet
j2f..	Sulindac
j2fy.	Sulindac 100mg tablet
j2f1.	Clinoril 100mg tablet
j2fz.	Sulindac 200mg tablet
j2f2.	Clinoril 200mg tablet
j2l..	Tenoxicam
j2l1.	Tenoxicam 20mg tablet
j2l2.	Mobiflex 20mg tablet
j2l5.	Tenoxicam 20mg effervescent tablet
j2l6.	Mobiflex 20mg effervescent tablet
j2l3.	Tenoxicam 20mg/sachet granules
j2l4.	Mobiflex Milk 20mg/sachet granules
j2l7.	Tenoxicam 20mg injection+diluent
j2l8.	Mobiflex vial 20mg injection+diluent
j2g..	Tiaprofenic acid
j2gx.	Tiaprofenic acid 200mg tablet
j2g1.	Surgam 200mg tablet
j2gy.	Tiaprofenic acid 300mg tablet
j2g2.	Surgam 300mg tablet
j2g5.	Tiaprofenic acid 300mg m/r capsule
j2g4.	Surgam SA 300mg m/r capsule
j2gz.	Tiaprofenic acid 300mg/sachet granules
j2g3.	Surgam 300mg/sachet granules
j2h..	Tolmetin
j2hy.	Tolmetin 200mg capsule
j2h2.	Tolectin 200mg capsule
j2hz.	Tolmetin 400mg capsule
j2h3.	Tolectin 400mg capsule
j2h1.	Tolectin DS 400mg capsules

READ_CD	DESCRIPTION (DMARD)
ej26.	Chloroquine phosphate 250mg tablets
ej25.	Nivaquine 272.5mg(200mg base)/5ml Injection (Aventis Pharma)
ejC..	Chloroquine phosphate 250mg tablets and Proguanil 100mg tablets

j54z.	Hydroxychloroquine 200mg tablets
ej24.	Nivaquine 68mg/5ml Oral solution (Aventis Pharma)
ej23.	Nivaquine 200mg Tablet (Aventis Pharma)
ej21.	Avloclor 250mg tablets (AstraZeneca UK Ltd)
ej31.	Plaquenil 200mg tablets (Sanofi)
j541.	Plaquenil 200mg tablets (Sanofi)
ej2w.	Chloroquine phosphate 80mg/5ml oral solution
ej22.	Malarivon 80mg/5ml syrup (Wallace Manufacturing Chemists Ltd)
j542.	Quinoric 200mg tablets (Bristol Laboratories Ltd)
h871.	Adalimumab 40mg injection
h873.	Adalimumab 40mg injection
h89w.	Enbrel 25mg powder and solvent for solution for injection vials (Pfizer Ltd)
h892.	Etanercept 25mg powder and solvent for solution for injection vials
h8Bz.	Infliximab 100mg powder for solution for infusion vials
h89z.	Enbrel 50mg powder and solvent for solution for injection vials (Wyeth Pharmaceuticals)
h8B1.	Remicade 100mg powder for solution for infusion vials (Merck Sharp & Dohme Ltd)
h872.	Humira 40mg Injection (Abbott Laboratories Ltd)
h874.	Humira 40mg Injection (Abbott Laboratories Ltd)
h891.	Etanercept 50mg powder and solvent for solution for injection vials
h893.	Etanercept 50mg injection solution
h895.	Etanercept 50mg injection solution
h89v.	Enbrel 25mg/0.5ml solution for injection pre-filled syringes (Pfizer Ltd)
h894.	Etanercept 25mg/0.5ml solution for injection pre-filled syringes
h89u.	Enbrel 50mg Solution for injection (Pfizer Consumer Healthcare Ltd)
h89x.	Enbrel 50mg Solution for injection (Pfizer Consumer Healthcare Ltd)
h89y.	Enbrel Paediatric 25mg powder and solvent for solution for injection vials (Pfizer Ltd)
h8G2.	Cimzia 200mg/1ml solution for injection pre-filled syringes (UCB Pharma Ltd)
h8G1.	Certolizumab pegol 200mg/1ml solution for injection pre-filled syringes
h71z.	Azathioprine 50mg powder for solution for injection vials
h71y.	Azathioprine 25mg tablets
h71x.	Azathioprine 50mg tablets
h712.	Imuran 25mg Tablet (Wellcome Medical Division)
h713.	Imuran 50mg Tablet (Wellcome Medical Division)
h711.	Azamune 50mg Tablet (Penn Pharmaceuticals Ltd)
h718.	Azathioprine 10mg tablets
h714.	Imuran 50mg powder for solution for injection vials (Aspen Pharma Trading Ltd)
h717.	Oprisine 50mg Tablet (Opus Pharmaceuticals Ltd)
h715.	Immunoprin 50mg tablets (Ashbourne Pharmaceuticals Ltd)
h716.	Berkaprine 50mg Tablet (Rorer Pharmaceuticals Ltd)
h719.	Imuran 10mg Tablet (Wellcome Medical Division)
hh11.	Rituximab 100mg/10ml solution for infusion vials
hh12.	Rituximab 500mg/50ml solution for infusion vials
h14..	Cyclophosphamide
h146.	Endoxana 10mg tablet

h141.	Cyclophosphamide 50mg tablet
h147.	Endoxana 50mg tablet
h142.	Cyclophosphamide 100mg injection (pdr for recon)
h148.	Endoxana 100mg injection (pdr for recon)
h143.	Cyclophosphamide 200mg injection (pdr for recon)
h14A.	Cyclophos 200mg injection (pdr for recon)
h149.	Endoxana 200mg injection (pdr for recon)
h144.	Cyclophosphamide 500mg injection (pdr for recon)
h14B.	Cyclophos 500mg injection (pdr for recon)
h145.	Cyclophosphamide 1g injection (pdr for recon)
h14C.	Cyclophos 1g injection (pdr for recon)
x00Nb	Cyclophosphamide 1g/50mL infusion
x00Na	Cyclophosphamide 2g/100mL infusion
x00NZ	Cyclophosphamide 4g/200mL infusion
h82A.	Neoral 25mg capsules (Novartis Pharmaceuticals UK Ltd)
h82C.	Neoral 100mg capsules (Novartis Pharmaceuticals UK Ltd)
h82x.	Ciclosporin 100mg/ml oral solution sugar free
h82D.	Neoral 100mg/ml oral solution (Novartis Pharmaceuticals UK Ltd)
h829.	Ciclosporin 50mg capsules
h826.	Ciclosporin 25mg capsules
h827.	Ciclosporin 100mg capsules
h824.	Sandimmun 25mg capsules (Novartis Pharmaceuticals UK Ltd)
h82B.	Neoral 50mg capsules (Novartis Pharmaceuticals UK Ltd)
h821.	Sandimmun 100mg/ml oral solution (Novartis Pharmaceuticals UK Ltd)
h825.	Sandimmun 100mg capsules (Novartis Pharmaceuticals UK Ltd)
h828.	Sandimmun 50mg capsules (Novartis Pharmaceuticals UK Ltd)
h82E.	Ciclosporin 10mg capsules
h82F.	Neoral 10mg capsules (Novartis Pharmaceuticals UK Ltd)
h822.	Sandimmun 50mg/ml Concentrate for solution for infusion (Novartis Pharmaceuticals UK Ltd)
h82y.	Ciclosporin 50mg/1ml solution for infusion ampoules
h82I.	Deximune 50mg capsules (Dexcel-Pharma Ltd)
h82J.	Deximune 100mg capsules (Dexcel-Pharma Ltd)
h82H.	Deximune 25mg capsules (Dexcel-Pharma Ltd)
h82z.	Ciclosporin 250mg/5ml solution for infusion ampoules
h82..	Ciclosporin product
h82G.	Sangcya 100mg/mL oral solution
h823.	Sandimmun 250mg/5mL oily infusion concentrate
h82K.	CAPIMUNE 25mg capsules
h82L.	CAPIMUNE 50mg capsules
h82M.	CAPIMUNE 100mg capsules
h82N.	CAPSORIN 25mg capsules
h82O.	CAPSORIN 50mg capsules
h82P.	CAPSORIN 100mg capsules
j513.	Myocrisin 10mg/0.5ml solution for injection ampoules (Sanofi)
j515.	Myocrisin 50mg/0.5ml solution for injection ampoules (Sanofi)

j514.	Myocrisin 20mg/0.5ml solution for injection ampoules (Sanofi)
j51z.	Sodium aurothiomalate 50mg/0.5ml solution for injection ampoules
j51x.	Sodium aurothiomalate 10mg/0.5ml solution for injection ampoules
j51y.	Sodium aurothiomalate 20mg/0.5ml solution for injection ampoules
x01J9	Hydroxychloroquine
ej3..	Hydroxychloroquine sulphate [anti malarial]
j54..	Hydroxychloroquine sulphate [anti- rheumatic]
h862.	Kineret 100mg/0.67ml solution for injection pre-filled syringes (Swedish Orphan Biovitrum Ltd)
h861.	Anakinra 100mg/0.67ml solution for injection pre-filled syringes
h8Fz.	Tocilizumab 80mg/4ml solution for infusion vials
h8Fy.	Tocilizumab 200mg/10ml solution for infusion vials
j59z.	Leflunomide 100mg tablets
j59x.	Leflunomide 10mg tablets
j59y.	Leflunomide 20mg tablets
j591.	Arava 10mg tablets (Sanofi)
j592.	Arava 20mg tablets (Sanofi)
j593.	Arava 100mg tablets (Sanofi)
h34..	METHOTREXATE
h341.	METHOTREXATE 2.5mg tablets
h342.	METHOTREXATE 10mg tablets
h343.	METHOTREXATE 2.5mg/1mL solution for injection
h344.	METHOTREXATE 5mg/2mL solution for injection
h345.	METHOTREXATE 25mg/1mL solution for injection
h346.	METHOTREXATE 50mg/2mL solution for injection
h347.	METHOTREXATE 100mg/4mL injection solution
h348.	METHOTREXATE 200mg/8mL solution for injection
h349.	METHOTREXATE 500mg/20mL solution for injection
h34A.	METHOTREXATE 15mg/1.5mL solution for injection prefilled syringe
h34B.	METHOTREXATE 20mg/2mL solution for injection prefilled syringe
h34C.	METHOTREXATE 25mg/2.5mL solution for injection prefilled syringe
h34D.	METOJECT 7.5mg/0.75mL solution for injection prefilled syringe
h34E.	METOJECT 10mg/1mL solution for injection prefilled syringe
h34F.	METOJECT 15mg/1.5mL solution for injection prefilled syringe
h34G.	METOJECT 20mg/2mL solution for injection prefilled syringe
h34H.	METOJECT 25mg/2.5mL solution for injection prefilled syringe
h34i.	EMTEXATE 1g/40mL solution for injection
h34j.	EMTEXATE 5g/200mL solution for injection
h34k.	EMTEXATE 1g/10mL solution for injection
h34L.	METOJECT 7.5mg/0.15mL solution for injection pfs
h34M.	METHOTREXATE 7.5mg/0.15mL solution for injection pfs
h34N.	METOJECT 10mg/0.2mL solution for injection prefilled syringe
h34O.	METHOTREXATE 10mg/0.2mL solution for injection pfs
h34P.	METOJECT 15mg/0.3mL solution for injection prefilled syringe
h34Q.	METHOTREXATE 15mg/0.3mL solution for injection pfs
h34R.	METOJECT 20mg/0.4mL solution for injection prefilled syringe

h34S.	METHOTREXATE 20mg/0.4mL solution for injection pfs
h34T.	METOJECT 25mg/0.5mL solution for injection prefilled syringe
h34U.	METHOTREXATE 25mg/0.5mL solution for injection pfs
h34V.	METOJECT 30mg/0.6mL solution for injection prefilled syringe
h34W.	METHOTREXATE 30mg/0.6mL solution for injection pfs
h34X.	EBETREX 7.5mg/0.75mL soln for injection prefilled syringe
h34Y.	EBETREX 10mg/1mL solution for injection prefilled syringe
h34Z.	EBETREX 15mg/1.5mL solution for injection prefilled syringe
h3G1.	EBETREX 20mg/1mL solution for injection prefilled syringe
h3G2.	EBETREX 25mg/1.25mL solution for injection prefilled syringe
h3G3.	EBETREX 30mg/1.5mL solution for injection prefilled syringe
h3G4.	METHOTREXATE 20mg/1mL solution for injection p/f syringe
h3G5.	METHOTREXATE 25mg/1.25mL solution for injection p/f syringe
h3G6.	METHOTREXATE 30mg/1.5mL solution for injection p/f syringe
h3G7.	METOJECT 12.5mg/0.25mL soln for injection prefilled syringe
h3G8.	METHOTREXATE 12.5mg/0.25mL solution for injection pfs
h3G9.	METOJECT 17.5mg/0.35mL soln for injection prefilled syringe
h3GA.	METHOTREXATE 17.5mg/0.35mL solution for injection pfs
h3GB.	METOJECT 22.5mg/0.45mL soln for injection prefilled syringe
h3GC.	METHOTREXATE 22.5mg/0.45mL solution for injection pfs
h3GD.	METOJECT 27.5mg/0.55mL soln for injection prefilled syringe
h3GE.	METHOTREXATE 27.5mg/0.55mL solution for injection pfs
j561.	Auranofin 3mg tablets
j562.	Ridaura Tiltab 3mg tablets (Astellas Pharma Ltd)
j521.	Penicillamine 50mg tablets
j523.	Penicillamine 250mg tablets
j522.	Penicillamine 125mg tablets
j525.	Distamine 125mg tablets (Alliance Pharmaceuticals Ltd)
j526.	Distamine 250mg tablets (Alliance Pharmaceuticals Ltd)
j524.	Distamine 50mg Tablet (Alliance Pharmaceuticals Ltd)
j528.	Pendramine 250mg Tablet (Viatris Pharmaceuticals Ltd)
j527.	Pendramine 125mg Tablet (Viatris Pharmaceuticals Ltd)
aa6z.	Sulfasalazine 3g/100ml enema
aa64.	Salazopyrin 3g/100ml Enema (Pharmacia Ltd)
aa62.	Salazopyrin EN-Tabs 500mg (Pfizer Ltd)
j551.	Salazopyrin EN-Tabs 500mg (Pfizer Ltd)
aa6v.	Sulfasalazine 500mg gastro-resistant tablets
aa61.	Salazopyrin 500mg Tablet (Pharmacia Ltd)
aa6y.	Sulfasalazine 500mg tablet
j55z.	Sulfasalazine 500mg suppositories
aa63.	Salazopyrin 500mg Suppository (Pharmacia Ltd)
aa6u.	Sulfasalazine 250mg/5ml oral solution
aa65.	Salazopyrin 250mg/5ml oral suspension (Pfizer Ltd)
aa66.	Sulfasalazine 500mg gastro-resistant tablets (Actavis UK Ltd)
	Sulazine EC 500mg tablets (Genesis Pharmaceuticals Ltd)
j552.	

READ_CD	DESCRIPTION (SICKNOTE)
9DK..	Sick note generated from secondary care done by GP practice
9C8..	Sickness notification of GP
9C83.	Sickness payment record
13O..	Sickness benefit
13O1.	Sickness benefit
13O2.	Statutory sick pay
9K8..	Sickness certificate

Supplementary Table S2: Definition of serious infections from secondary care records using the International Classification of Diseases- 10th Revision

ICD-10 Codes	Upper respiratory tract infections
J02.0	Streptoccocal sore throat
J00	Acute nasopharyngitis (common cold)
J01.90	Acute sinusitis, unspecified
J02.9	Acute phartngitis
J03.90	Acute tonsilitis
J06.9	Acute upper respiratory infections of unspecified site
J31.0	Chronic rhinitis
J32.9	Unspecified sinusitis (chronic)
R05	Cough
	**Lower respiratory tract infections**
A37.90	Whooping cough, unpecified organism
B97.4	Respiratory syncytial virus
J41.0	Simple chronic bronchitis
J41.1	Micropurulent chronic bronchitis
J41.8	Mixed simple and micropurulent chronic bronchitis
J42.0	Unspecified chronic bronchitis
J14	Pneumonia due to Haemophilus influenzae
J15.0	Pneumonia due to Klebsiella pneumoniae
J15.1	Pneumonia due to Pseudomonas
J15.2	Pneumonia due to staphylococcus
J15.3	Pneumonia due to streptococcus, group B
J15.4	Pneumonia due to other streptococci
J15.5	Pneumonia due to Escherichia coli
J15.6	Pneumonia due to other aerobic Gram-negative bacteria
J15.7	Pneumonia due to Mycoplasma pneumoniae
J15.8	Other bacterial pneumonia
J15.9	Bacterial pneumonia, unspecified
J18.0	Bronchopneumonia, unspecified
J18.1	Lobar pneumonia, unspecified
J18.2	Hypostatic pneumonia, unspecified
J18.8	Other pneumonia, organism unspecified
J18.9	Pneumonia, unspecified
R09.1	Pleurisy
	**Tuberculosis**
A15.0	Tuberculosis of lung, confirmed by sputum microscopy with or without culture
A15.1	Tuberculosis of lung, confirmed by culture only
A15.2	Tuberculosis of lung, confirmed histologically
A15.3	Tuberculosis of lung, confirmed by unspecified means
A15.4	Tuberculosis of intrathoracic lymph nodes, confirmed bacteriologically and histologically
A15.5	Tuberculosis of larynx, trachea and bronchus, confirmed bacteriologically and histologically

A15.6	Tuberculous pleurisy, confirmed bacteriologically and histologically
A15.7	Primary respiratory tuberculosis, confirmed bacteriologically and histologically
A15.8	Other respiratory tuberculosis, confirmed bacteriologically and histologically
A15.9	Respiratory tuberculosis unspecified, confirmed bacteriologically and histologically
A16.2	Tuberculosis of lung, without mention of bacteriological or histological confirmation
A16.3	Tuberculosis of intrathoracic lymph nodes, without mention of bacteriological or histological confirmation
A16.4	Tuberculosis of larynx, trachea and bronchus, without mention of bacteriological or histological confirmation
A16.5	Tuberculous pleurisy, without mention of bacteriological or histological confirmation
A16.7	Primary respiratory tuberculosis without mention of bacteriological or histological confirmation
A16.8	Other respiratory tuberculosis, without mention of bacteriological or histological confirmation
A16.9	Respiratory tuberculosis unspecified, without mention of bacteriological or histological confirmation
A17.0	Tuberculous meningitis
A17.1	Meningeal tuberculoma
A17.8	Other tuberculosis of nervous system
A17.9	Tuberculosis of nervous system, unspecified
A18.0	Tuberculosis of bones and joints
A18.1	Tuberculosis of genitourinary system
A18.2	Tuberculous peripheral lymphadenopathy
A18.3	Tuberculosis of intestines, peritoneum and mesenteric glands
A18.4	Tuberculosis of skin and subcutaneous tissue
A18.5	Tuberculosis of eye
A18.6	Tuberculosis of ear
A18.7	Tuberculosis of adrenal glands
A18.8	Tuberculosis of other specified organs
A19.0	Acute miliary tuberculosis of a single specified site
A19.1	Acute miliary tuberculosis of multiple sites
A19.2	Acute miliary tuberculosis, unspecified
A19.8	Other miliary tuberculosis
A19.9	Miliary tuberculosis, unspecified
	**GI infections**
A03.0	Shigellosis due to Shigella dysenteriae
A03.1	Shigellosis due to Shigella flexneri
A03.2	Shigellosis due to Shigella boydii
A03.3	Shigellosis due to Shigella sonnei
A03.8	Other shigellosis
A03.9	Shingellosis, unspecified
A07.1	Giardiasis
A00.0	Cholera due to Vibrio cholerae 01, biovar cholerae

A00.1	Cholera due to Vibrio cholerae 01, biovar eltor
A00.9	Cholera, unspecified
A01.0	Typhoid fever
A01.1	Paratyphoid fever A
A01.2	Paratyphoid fever B
A01.3	Paratyphoid fever C
A01.4	Paratyphoid fever, unspecified
A09.0	Other and unspecified gastroenteritis and colitis of infectious origin
A09.9	Gastroenteritis and colitis of unspecified origin
A04.0	Enteropathogenic Escherichia coli infection
A04.1	Enterotoxigenic Escherichia coli infection
A04.2	Enteroinvasive Escherichia coli infection
A04.3	Enterohaemorrhagic Escherichia coli infection
A04.4	Other intestinal Escherichia coli infections
A04.5	Campylobacter enteritis
A04.6	Enteritis due to Yersinia enterocolitica
A04.7	Enterocolitis due to Clostridium difficile
A04.8	Other specified bacterial intestinal infections
A04.9	Bacterial intestinal infection, unspecified
B98.0	Helicobacter pylori [H.pylori] as the cause of diseases classified to other chapters
	**Meningitis**
A39.0	Meningococcal meningitis
A39.1	Waterhouse-Friderichsen syndrome
A39.2	Acute meningococcaemia
A39.3	Chronic meningococcaemia
A39.4	Meningococcaemia, unspecified
A39.5	Meningococcal heart disease
A39.8	Other meningococcal infections
A39.9	Meningococcal infection, unspecified
	**Sepsis**
A40.0	Sepsis due to streptococcus, group A
A40.1	Sepsis due to streptococcus, group B
A40.2	Sepsis due to streptococcus, group D
A40.3	Sepsis due to Streptococcus pneumoniae
A40.8	Other streptococcal sepsis
A40.9	Streptococcal sepsis, unspecified
A41.0	Sepsis due to Staphylococcus aureus
A41.1	Sepsis due to other specified staphylococcus
A41.2	Sepsis due to unspecified staphylococcus
A41.3	Sepsis due to Haemophilus influenzae
A41.4	Sepsis due to anaerobes
A41.5	Sepsis due to other Gram-negative organisms
A41.8	Other specified sepsis
A41.9	Sepsis, unspecified
T81.44%	Sepsis following procedure

	Genitourinary infections
N10	Acute tubulo-interstitial nephritis
N11.0	Nonobstructive reflux-associated chronic pyelonephritis
N11.1	Chronic obstructive pyelonephritis
N11.8	Other chronic tubulo-interstitial nephritis
N11.9	Chronic tubulo-interstitial nephritis, unspecified
N12	Tubulo-interstitial nephritis, not specified as acute or chronic
N16.0	Renal tubulo-interstitial disorders in infectious and parasitic diseases classified elsewhere
N30.0	Acute cystitis
N30.1	Interstitial cystitis (chronic)
N30.2	Other chronic cystitis
N30.3	Trigonitis
N30.4	Irradiation cystitis
N30.8	Other cystitis
N30.9	Cystitis, unspecified
N39.0	Urinary tract infection, site not specified
	**Joint infection**
M00%	Pyogenic arthritis
M01%	Direct infections of joint in infectious and parasitic diseases classified elsewhere
M02%	Postinfective and reactive arthropathies
	**Skin infection**
B95.6%	Staphylococcus aureus as the cause of diseases classified elsewhere
L03.0%	Cellulitis and acute lymphangitis of finger and toe
L03.1%	Cellulitis and acute lymphangitis of other parts of limb
L03.2%	Cellulitis and acute lymphangitis of face and neck
L03.3%	Cellulitis and acute lymphangitis of trunk
L03.8%	Cellulitis and acute lymphanitis of other sites
L03.9%	Cellulitis and acute lymphanitis, unspecified
L08.8%	Other specified local infection of the skin and subcutaneous tissue
L08.9	Local infection of the skin and subcutaneous tissue, unspec

Supplementary Table S3: Orthopaedic surgery as indicated by OPCS-4 intervention and procedure codes from secondary care datasets

OPCS-4 Code	OPCS-4 Term	
**W37**	**Monk total replacement of hip joint using cement**	
W370	Convrs from cement tot hip rep	Conversion from cemented total hip replacement
W371	Primary cemented tot hip repl	Primary cemented total hip replacement
W372	Conv to cemented tot hip repl	Conversion to cemented total hip replacement
W373	Revsion cemented total hip rep	Revision cemented total hip replacement
W378	Tot prosth repl hip + cem OS	Total prosthetic replacement of hip joint using cement OS
W379	Tot prosth repl hip + cem NOS	Total prosthetic replacement of hip joint using cement NOS
**W38**	**Lord total hip replace no cem**	**Lord total replacement of hip joint not using cement**
W380	Conv from uncement tot hip rep	Conversion from uncemented total hip replacement
W381	Pry uncmnt tot hip replacement	Primary uncemented total hip replacement
W382	Cnvrs to uncmnt tot hip rplcmn	Conversion to uncemented total hip replacement
W383	Revisn uncemented tot hip rep	Revision uncemented total hip replacement
W388	Tot prosth repl hip no cem OS	Total prosthetic replacement hip joint not using cement OS
W389	Tot prosth repl hip no cem NOS	Total prosthetic replacement hip joint not using cement NOS
**W39**	**Other total prosth repl hip**	**Other total prosthetic replacement of hip joint**
W390	Removal prev tot hip repl NEC	Removal previous total prosthetic replacement hip joint NEC
W391	Pry hybrid tot hip rplcmnt NEC	Primary hybrid total hip replacement NEC
W392	Cnvrs to hbrd tot hip rplc NEC	Conversion to hybrid total hip replacement NEC
W393	Revisn hybrid tot hip rep NEC	Revision hybrid total hip replacement NEC
W394	Attntn to tot hip rplcmnt NEC	Attention to total hip replacement NEC
W398	Total prosthet replace hip OS	Other specified total prosthetic replacement of hip joint
W399	Total prosthet replace hip NOS	Total prosthetic replacement of hip joint NOS
**W40**	**Shiers total knee replacement**	**Shiers total replacement of knee joint using cement**
**W40-W87**	**Knee joint operations**	
W400	Con from cemented tot knee rep	Conversion from cemented total knee replacement
W401	Pry cemented total knee replac	Primary cemented total knee replacement
W402	Conv to cemented tot knee repl	Conversion to cemented total knee replacement
W403	Revision cemented tot knee rep	Revision cemented total knee replacement
W408	Tot prosth repl knee + cem OS	Total prosthetic replacement of knee joint using cement OS
W409	Tot prosth repl knee + cem NOS	Total prosthetic replacement of knee joint using cement NOS
W41	Arthroplasty knee no cement	Arthroplasty of knee joint not using cement

W410	Removal prv uncem tot knee rep	Removal previous uncemented total prosthet replacement knee
W411	Primary uncmnt tot knee repl	Primary uncemented total knee replacement
W412	Cnvrs to uncmnt tot knee repl	Conversion to uncemented total knee replacement
W413	Revision uncmnt tot knee repl	Revision uncemented total knee replacement
W418	Tot pros repl knee no cem OS	Total prosthetic replacement knee joint not using cement OS
W419	Tot pros repl knee no cem NOS	Total prosthetic replacement knee joint not using cement NOS
**W42**	**Other arthroplasty knee joint**	**Other arthroplasty of knee joint**
W420	Cnv fr hybrd tot knee repl NEC	Conversion from hybrid total knee replacement NEC
W421	Pry hybrid tot knee repl NEC	Primary hybrid total knee replacement NEC
W422	Con to hybrid tot knee rep NEC	Conversion to hybrid total knee replacement NEC
W423	Revi hybrid tot knee repl NEC	Revision of hybrid total knee replacement NEC
W424	Attention to tot knee repl NEC	Attention to total knee replacement NEC
W428	Other tot prosth knee repl OS	Other total prosthetic replacement of knee joint OS
W429	Other tot prosth knee rep NOS	Other total prosthetic replacement of knee joint NOS
**W43**	**Prosth cmntd tot shldr rplcmnt**	**Prosthetic cemented total shoulder replacement**
**W43-W45**	**Elbow joint operations**	
**W43-W92**	**Other joint operations**	
W430	Cnvr fr cmnt tot shldr rplcmnt	Conversion from cemented total shoulder replacement
W431	Pry cmntd tot shldr replacmnt	Primary cemented total shoulder replacement
W432	Cnvr to cmntd tot shldr rplcmn	Conversion to cemented total shoulder replacement
W433	Rvsn cmntd tot shldr rplcmnt	Revision cemented total shoulder replacement
W438	Tot pros repl oth joint+cem OS	Total prosthetic replacement of other joint using cement OS
W439	Tot pr repl oth joint+cem NOS	Total prosthetic replacement of other joint using cement NOS
**W44**	**Prsth uncmnt tot shldr rplcmnt**	**Prosthetic uncemented total shoulder replacement**
W440	Cnvr fr uncmnt tot shldr rplcm	Conversion from uncemented total shoulder replacement
W441	Pry uncmnt tot shldr replcmnt	Primary uncemented total shoulder replacement
W442	Cnvr to uncmnt tot shldr rplcm	Conversion to uncemented total shoulder replacement
W443	Rvsn uncmntd tot shldr rplcmnt	Revision uncemented total shoulder replacement
W448	Other joint repl no cement OS	Other total prosthet replacem oth joint not using cement OS
W449	Other joint repl no cement NOS	Other total prosthet replacem oth joint not using cement NOS
**W45**	**Prosth hybrid tot shldr rplcmn**	**Prosthetic hybrid total shoulder replacement**
W450	Cnvr fr hybrid tot shldr rplcm	Conversion from hybrid total shoulder replacement
W451	Pry hybrid tot shldr rplcment	Primary hybrid total shoulder replacement
W452	Cnvr to hybrd tot shldr rplcmn	Conversion to hybrid total shoulder replacement
W453	Rvsn hybrid total shldr rplcmn	Revision hybrid total shoulder replacement
W454	Atten tot prosth joint rep NEC	Attention to total prosthetic replacement of joint NEC

W454/Y037	Rem prosth joint (no replace)	Removal prosthesis from joint (no replacement)
W458	Other tot prosth repl joint OS	Other specified other total prosthetic replacement of joint
W459	Other tot prosth rep joint NOS	Other total prosthetic replacement of joint NOS
**W46**	**Austin-Moore hemiarthropl hip using cement**	**Austin - Moore hemiarthroplasty of hip joint**
W460	Removal prev cem repl hd femur replacement head femur	Removal previous cemented prosthetic
W461	Pry cmntd hemiarthroplasty hip	Primary cemented hemiarthroplasty of hip
W462	Cnvrs to cmnt hemiarthrpls hip hip	Conversion to cemented hemiarthroplasty of
W463	Rvsn cmntd hemiarthroplsty hip	Revision cemented hemiarthroplasty of hip
W468	Oth sp prsth cmntd hemiart hip hemiarthroplasty of hip	Other specified prosthetic cemented
W469	Prsth cmnt hemiarthrpl hip NOS NOS	Prosthetic cemented hemiarthroplasty of hip
**W47**	**Prsth uncmnt hemiarthrpl hip**	**Prosthetic uncemented hemiarthroplasty of hip**
W470	Cnvr fr uncmnt hemiarthrpl hip hemiarthroplasty of hip	Conversion from uncemented
W471	Pry uncmntd hemiarthropl hip	Primary uncemented hemiarthroplasty of hip
W472	Cnvrs to uncmnt hemiarthrp hip of hip	Conversion to uncemented hemiarthroplasty
W473	Rvsn uncmnt hemiarthrpl hip	Revision uncemented hemiarthroplasty of hip
W478	Other pros uncem hemiarthr hip hemiarthroplasty hip	Other specified prosthetic uncemented
W479	Prsth uncmn hemiarthrp hip NOS hip NOS	Prosthetic uncemented hemiarthroplasty of
**W48**	**Other arthroplasty head femur**	**Other arthroplasty of head of femur**
W480	Cnvr fr prev hemiarthr hip NEC of hip NEC	Conversion from previous hemiarthroplasty
W481	Pry prosth hemiarthrpl hip NEC NEC	Primary prosthetic hemiarthroplasty of hip
W482	Cnvr to prsth hemiarth hip NEC hip NEC	Conversion to prosthetic hemiarthroplasty of
W483	Rvsn prsth hemiarthrpl hip NEC NEC	Revision of prosthetic hemiarthroplasty of hip
W484	Atn to prsth hemiarthr hip NEC hip NEC	Attention to prosthetic hemiarthroplasty of
W488	Oth sp oth prsth hemiarthr hip	Other specified other prosthetic hemiarthroplasty of hip
W489	Oth prosth hemiarthrpl hip NOS	Other prosthetic hemiarthroplasty of hip NOS
**W49**	**Pros replacem head hum + cemnt using cement**	**Prosthetic replacement of head of humerus**
**W49-W51**	**Shoulder joint operations**	
W490	Cnvrsn fr cmnt hemiarthr shldr of shoulder	Conversion from cemented hemiarthroplasty
W491	Primary cmntd hemiarthr shldr shoulder	Primary cemented hemiarthroplasty of

W492	Cnvrs to cmntd hemiarthr shldr	Conversion to cemented hemiarthroplasty of shoulder
W493	Rvsn cmntd hemiarthr shldr	Revision cemented hemiarthroplasty of shoulder
W498	Oth spec cmntd hemiarthr shldr	Other specified cemented hemiarthroplasty of shoulder
W499	Prosth cmnt hemiarth shldr NOS	Prosthetic cemented hemiarthroplasty of shoulder NOS
**W50**	**Prosth uncmntd hemiarthr shldr**	**Prosthetic uncemented hemiarthroplasty of shoulder**
W500	Cnvr fr uncmnt hemiarth shldr	Conversion from uncemented hemiarthroplasty of shoulder
W501	Primary uncmnt hemiarthr shldr	Primary uncemented hemiarthroplasty of shoulder
W502	Cnvr to uncmnt hemiarthr shldr	Conversion to uncemented hemiarthroplasty of shoulder
W503	Rvsn uncmntd hemiarthr shldr	Revision uncemented hemiarthroplasty of shoulder
W508	Oth spec uncmnt hemiarth shldr	Other specified uncemented hemiarthroplasty of shoulder
W509	Pros uncmntd hemiart shldr NOS	Prosthetic uncemented hemiarthroplasty of shoulderNOS
**W51**	**Other prosth repl head humerus**	**Other prosthetic replacement of head of humerus**
W510	Conv from hyb hemrth shoul NEC	Conversion from hemiarthroplasty of shoulder NEC
W511	Prim hybr hemiarth should NEC	Primary hybrid hemiarthroplasty of shoulder NEC
W512	Conv-hybr hemiarth should NEC	Conversion to hybrid hemiarthroplasty of shoulder NEC
W513	Revis hyb hemiarth should NEC	Revision hybrid hemiarthroplasty of shoulder NEC
W514	Atten replace head humerus NEC	Attention to prosthetic replacement of head of humerus NEC
W518	Other prosth repl head hum OS	Other prosthetic replacement of head of humerus OS
W519	Oth prsth replcmnt hd hum NOS	Other prosthetic replacement of head of humerus NOS
**W52**	**Cmntd unicmprtmntl kn rplcmnt**	**Cemented unicompartmental knee replacement**
W520	Cnvr fr cmnt unicmprt kn rplcm	Conversion from cemented unicompartmental knee replacement
W521	Pry cmntd unicmprtmn kn rplcmn	Primary cemented unicompartmental knee replacement
W522	Cnvr to cmnt unicmpr kn rplcmn	Conversion to cemented unicompartmental knee replacement
W523	Rvsn cmnt unicmprtmn kn rplcmn	Revision cemented unicompartmental knee replacement
W528	Prosth repl oth artic + cem OS	Prosthetic replacement articulation oth bone using cement OS
W529	Pros repl oth artic + cem NOS	Prosthetic replacement articulatn oth bone using cement NOS

**W53**	**Uncmnt unicmprtmnt kn rplcmnt replacement**	**Uncemented unicompartmental knee**
W530	Cnvr fr uncmn unicmprt kn rplc unicompartmental knee replacement	Conversion from uncemented
W531	Prmy uncmnt unicmprt kn rplcmn replacement	Primary uncemented unicompartmental knee
W532	Cnvr to uncmn unicmprt kn rplc unicompartmental knee replacement	Conversion to uncemented
W533	Rvsn uncmnt unicmprtm kn rplcm knee replacement	Revision uncemented unicompartmental
W538	Prosth repl articul no cem OS cement OS	Prosthet replacement articulat oth bone not using
W539	Prosth repl articul no cem NOS cement NOS	Prosthet replacement articulat oth bone not using
**W54**	**Hybrid unicomprtmnt kn rplcmnt**	**Hybrid unicompartmental knee replacement**
W540	Cnvr fr hybrd unicmprt kn rplc replacement	Conversion from hybrid unicompartmental knee
W541	Pry hybrid unicmprtm kn rplcmn replacement	Primary hybrid unicompartmental knee
W542	Cnvr to hybrd unicmpr kn rplcm replacement	Conversion to hybrid unicompartmental knee
W543	Rvsn hybrd unicmprtmt kn rplcm replacement	Revision hybrid unicompartmental knee
W544	Atten repl articul bone NEC	Attention to prosthetic replacement of articulation NEC
W548	Other repl artic oth bone OS bone OS	Other prosthetic replacement of articulation of oth
W549	Other repl artic oth bone NOS bone NOS	Other prosthetic replacement of articulation of oth
**W55**	**Prosth interposit arthroplasty**	**Prosthetic interposition arthroplasty**
W550	Con proth intrpstn arthrpls arthroplasty	Conversion from previous prosth interposition
W551	Pry proth interpstn arthrplsty	Primary prosthetic interposition arthroplasty
W552	Rvsn proth interpstn arthrplsy	Revision of prosthetic interposition arthroplasty
W553	Cnvr to prth intrpstn arthrpls	Conversion to prosthetic interposition arthroplasty
W554	Atn prth intrpstn arthrpls NEC	Attention to prosthetic interposition arthroplasty NEC
W558	Oth sp prth intrpstn arthrplsy	Other specified prosthetic interposition arthroplasty
W559	Proth intrpstn arthroplast NOS	Prosthetic interposition arthroplasty NOS
**W56**	**Other interposit arthroplasty**	**Other interposition arthroplasty**
W560	Con prev intrpstn arthrpls NEC NEC	Conversion from previous interposition arthroplasty
W561	Prim interpos arth MTP joint NEC	Primary interposit arthroplasty metatarsophalang joint
W562	Pry interpstn arthroplasty NEC	Primary interposition arthroplasty NEC
W563	Rvsn of intrpstn arthrplst NEC	Revision of interposition arthroplasty NEC
W564	Cnvr to intrpstn arthrplst NEC	Conversion to interposition arthroplasty NEC
W568	Oth sp interpstn recnstrctn jt	Other specified interposition reconstruction of joint
W569	Interposit reconstr joint NOS	Interposition reconstruction of joint NOS

**W57**	**Excision arthroplasty**	
W570	Cnvr fr prev excsn arthroplsty	Conversion from previous excision arthroplasty
W571	Prim exc arthropl 1st MTP join joint	Primary excision arthroplasty 1st metatarsophalangeal
W572	Pry excision arthroplasty NEC	Primary excision arthroplasty NEC
W572/Z843	Excision arthroplasty of hip	
W573	Rvsn of excision arthroplasty	Revision of excision arthroplasty
W574	Cnvrsn to excisn arthroplasty	Conversion to excision arthroplasty
W578	Oth sp excsn reconstrctn of jt	Other specified excision reconstruction of joint
W579	Exc reconstruction joint NOS	Excision reconstruction of joint NOS
**W58**	**Other arthroplasty**	
W580	Conv fr prev resurf arthroplas joint	Conversion from previous resurfacing arthroplasty of
W581	Prim resurfacing arthroplasty	Primary resurfacing arthroplasty of joint
W582	Rvsn of resurfcng arthroplasty	Revision of resurfacing arthroplasty
W588	Reconstruct joint c free flap	Reconstruction of joint with free flap
W589	Other reconstruction joint NOS	Other reconstruction of joint NOS
**W59**	**Fusion of other toe joint**	
**W59 -W79**	**Foot joint operations**	
W591	Pry arthrodesis 1st MTPJ & rep MTPJ	Primary arthrodesis 1st MTPJ & replace lesser
W592	Pry arthrodesis 1st MTPJ & exc lesser MTPJ	Primary arthrodesis 1st MTPJ & excision
W593	Fusion of first MTP joint	Fusion of first metatarsophalangeal joint of toe
W594	Pry arthrdsis IPJ great toe	Primary arthrodesis interphalangeal joint of great toe
W595	Pry arthrdsis IPJ oth toe NEC NEC	Primary arthrodesis of interphalangeal joint other toe
W596	Revision arthrdsis toe joint	Revision arthrodesis of toe joint
W598	Fusion of first MTP joint OS	Other specified fusion of first metatarsophalangeal joint
W599	Fusion of other toe joint NOS	
**W60**	**Fuse joint extraart bone graft**	**Fusion of other joint and extraarticular bone graft**
W600	Cnvrs fr extrartic arthrod NEC	Conversion from extraarticular arthrodesis NEC
W601	Pry extraart arthrodsis NEC	Primary extraarticular arthrodesis of joint NEC
W602	Rvsn extrartic arthrodesis NEC	Revision extraarticular arthrodesis NEC
W603	Conv to extrart arthrodsis NEC	Conversion to extraarticular arthrodesis NEC
W608	Fuse joint & extraart graft OS	Fusion of joint and extraarticular bone graft OS
W609	Fuse joint & extraart graf NOS	Fusion of joint and extraarticular bone graft NOS
**W61**	**Other fuse joint & artic graft**	**Fusion of other joint and other articular bone graft**
W610	Cnv fr prev intraart arthrdsis	Conversion from previous intraarticular arthrodesis
W611	Prim arthrod & artic graft NEC	Primary arthrodesis and articular bone graft NEC
W612	Rev arthrod & artic graft NEC	Revision of arthrodesis and articular bone graft NEC
W613	Conv to arthrod artic graf NEC	Conversion to arthrodesis and articular bone graft NEC
W618	Fuse joint & artic graft OS	Other specified fusion of joint and articular bone graft
W619	Fuse joint & artic graft NOS	Fusion of joint and articular bone graft NOS
**W62**	**Other primary fusion of joint**	
W621	Prim arthrodes & int fixat NEC	Primary arthrodesis and internal fixation of joint NEC
W622	Prim arthrodes & ext fixat NEC	Primary arthrodesis and external fixation of joint NEC
W628	Other primary arthrodesis OS	Other specified other primary fusion of joint

W629	Simple arthrodesis	
**W63**	**Revisional fusion of joint**	
W631	Revis arthrodes int fixat NEC	Revision of arthrodesis and internal fixation NEC
W632	Revis arthrodes ext fixat NEC	Revision of arthrodesis and external fixation NEC
W638	Revisional fusion of joint OS	Other specified revisional fusion of joint
W639	Revision of arthrodesis NEC	
**W64**	**Conversion to arthrodesis NEC**	
W640	Conversion from prev arthrodes	Conversion from previous arthrodesis NEC
W641	Conv to arthrod & int fix NEC	Conversion to arthrodesis and internal fixation NEC
W642	Conv to arthrod & ext fix NEC	Conversion to arthrodesis and external fixation NEC
**V22**	**Prim decompress cervical spine**	**Primary decompression operation on cervical spine**
V22-V54	Cerv and thorac spine ops OS	Other specified operations on cervical or thoracic spine
V221	Pry ant dcmpr cx spn crd+fsn	Primary ant decompression of cervical spinal cord+fusion
V222	Pry ant dcmprs cx spn crd NEC	Primary anterior decompression of cervical spinal cord NEC
V223	Prim foraminotomy cerv spine	Primary foraminotomy of cervical spine
V228	Prmry post decomprsn cerv cord	Primary posterior decompression cervical cord
V229	Prim decomp cervical spine NOS	Primary decompression operation on cervical spine NOS
**V23**	**Revis decomp cervical spine**	**Revisional decompression operations on cervical spine**
V231	Rev ant dcmpr crv sp crd+fsn	Revisional ant decompression op cervicl spinal cord+fusion
V232	Rev ant decomp cerv cord NEC	Revisional anterior decompression cervical spinal cord NEC
V233	Revis foraminotomy cerv spine	Revisional foraminotomy of cervical spine
V238	Rev post decomprsn cerv cord	Revision posterior decompression cervical cord
V239	Rev decomp cervical spine NOS	Revisional decompression of cervical spine NOS
**V24**	**Decompress thoracic spine NEC**	**Decompression of thoracic spine NEC**
V241	Prim decompr fusion thor spine	Primary decompress thoracic spinal cord fusion thorac spine
V242	Prim decompress thor spine NEC	Primary decompression of thoracic spine NEC

V243	Rvsnal post decomprs thor dsc	Revisional posterior decompression of thoracic disc
V248	Decompression thorac spine OS	Other specified decompression of thoracic
	spine	
V249	Prmry post decompr thorac disc	Primary posterior decompression of thoracic
	disc	
**V25**	**Primary decomp lumbar spine op**	**Primary decompression operations on**
	**lumbar spine**	
V25-V39	Lumbar spinal cord operations	
V25-V54	Lumbar spine operations OS	Other specified operations on lumbar spine
V251	Pry extn pst dcmprs lmb sp+fsn	Primary extndd post decompression lumbar
	spine and fusion	
V252	Pry extnd pst dcmpr lmb sp NEC	Primary extended posterior decompression
	lumbar spine NEC	
V253	Prmy post dcmprsn lmbr spn+fsn	Primary posterior decompression lumbar
	spine and fusion	
V254	Prmy post dcmprsn lmbr spine	Primary posterior decompression lumbar spine
V255	Prim post decompr LS cord NEC	Primary posterior decompression of lumbar
	spinal cord NEC	
V256	Prmy foraminotomy lmbr spine	Primary foraminotomy of lumbar spine
V258	Prim decompr op lumb spine OS	Primary decompression operation on lumbar
	spine OS	
V259	Prim decompr op lumb spine NOS	Primary decompression operation on lumbar
	spine NOS	
**V26**	**Revis decompr ops lumbar spine**	**Revisional decompression operations on**
	**lumbar spine**	
V261	Rev extnd pst dcmpr lmb sp+fsn	Revision extndd post decompression lumbar
	spine and fusion	
V262	Rev extnd pst dcmpr lmb sp NEC	Revision extended posterior decompression
	lumbar spine NEC	
V263	Rvsn post dcmprsn lmbr spn+fsn	Revision posterior decompression lumbar
	spine and fusion	
V264	Rvsn post dcmprsn lmbr spine	Revision posterior decompression lumbar spine
V265	Revis post decomp LS cord NEC	Revisional posterior decompression of lumbar
	spinal cord NEC	
V266	Rvsnal foraminotomy lmbr spine	Revisional foraminotomy of lumbar spine
V268	Revis decompr op lumbar sp OS	Revisional decompression operation on
	lumbar spine OS	
V269	Revis decompr op lumb sp NOS	Revisional decompression operation on
	lumbar spine NOS	
**V27**	**Decompress unspecified spine**	**Decompression operations on unspecified**
	**spine**	
V27-V54	Oth spine operations,site unsp	Other spine operations, site unspecified

V271	Prim decomp sp cord & fuse NEC	Primary decompression of spinal cord & fusion spine jnt NEC
V272	Prim decompr spinal cord NEC	Primary decompression of spinal cord NEC
V273	Revis decompr spinal cord NEC	Revisional decompression of spinal cord NEC
V278	Decompression of spine OS	Other specified decompression of spine
V279	Decompression of spine NOS	
**V29**	**Primary excis cervical IV disc**	**Primary excision of cervical intervertebral disc**
**V29-V54**	**Cerv+thoracic spine ops**	**Cervical and thoracic spine operations**
V291	Pry laminec excis cerv IV disc	Primary laminectomy excision of cervical intervert disc
V292	1st hemilaminec cerv IV disc	Primary hemilaminectomy excision of cervical IV disc
V293	1st fenestration cerv IV disc	Primary fenestration excision of cervical intervert disc
V294	Badgeley ant fusion cerv spine	Badgeley anterior fusion of cervical spine
V295	1st ant excis cervic disc NEC	Primary anterior excision of cervical intervertebr disc NEC
V296	1st cervical microdiscectomy	Primary microdiscectomy of cervical intervertebral disc
V298	Primary excis cervical disc OS	Primary excision of cervical intervertebral disc OS
V299	1st post exc cervical IV disc	Primary posterior excision of cervical intervertebral disc
**V30**	**Revis cervical disc excis ops**	**Revisional excision of cervical intervertebral disc ops**
V301	Revis laminec exc cerv IV disc	Revisional laminectomy excision of cervical intervert disc
V302	Rev hemilaminect exc cerv disc	Revisional hemilaminectomy excision cervical intervert disc
V303	Rev fenestration exc cerv disc	Revisional fenestration excision of cervical intervert disc
V304	Rvsn ant excsn cerv disc+fusn	Revision anterior excision cervical disc and fusion
V305	Revis ant excis cerv disc NEC	Revisional anterior excision cervical intervert disc NEC
V306	Revis cervical microdiscectomy	Revisional microdiscectomy of cervical intervertebral disc
V308	Revis post excis cerv disc NEC	Revisional posterior excision of cervical intervert disc NEC
V309	Revis cervic IV disc excis NOS	Revisional excision of cervical intervertebral disc NOS
**V31**	**Excision of thoracic disc NEC**	**Excision of thoracic intervertebral disc NEC**

V311	Prmry ant excsn thor disc+fsn	Primary anterior excision thoracic disc and fusion
V312	Prim ant/lat exc thor disc NEC	Primary anterolateral excision thoracic intervert disc NEC
V313	Prim costotransversect th disc	Primary costotransversectomy of thoracic intervertebral disc
V318	Prim excis thoracic disc OS	Primary excision of thoracic intervertebral disc OS
V319	Prim excis thoracic disc NOS	Primary excision of thoracic intervertebral disc NOS
**V32**	**Rvsn excs thr intrvrt dsc NEC**	**Revisional excision of thoracic intervertebral disc NEC**
V321	Rvsn ant excsn thor disc+fsn	Revision anterior excision thoracic disc and fusion
V322	Rev AL excis thoracic disc NEC	Revisional anterolateral excision thorac intervert disc NEC
V323	Rev costotransversec thor disc	Revisional costotransversectomy thoracic intervertebral disc
V328	Rvsn decomprs+fsn thorac spn	Revision decompression and fusion thoracic spine
V329	Revis excis thoracic disc NOS	Revisional excision thoracic intervertebral disc NOS
**V33**	**Primary lumbar discectomy**	
V331	Prim laminect exc lumbar disc	Primary laminectomy excision of lumbar intervertebral disc
V332	Prim fenestration lumbar disc	Primary fenestration of lumbar intervertebral disc
V333	Prmy ant exc lmbr disc+fusion	Primary anterior excision of lumbar disc and fusion
V334	Prmy ant exc lmbr disc NEC	Primary anterior excision of lumbar disc NEC
V335	Prmy ant exc lmbr dsc+pst fsn	Primary anterior excision of lumbar disc and posterior fusn
V336	Pry ant exc lmb dsc+pst instrm	Primary ant excision lumbar disc+post instrumentation
V337	Primary lumbar microdiscectomy	
V338	Prmy post excision lumbar disc	Primary posterior excision of lumbar disc
V339	Primary lumbar discectomy NOS	Primary excision of lumbar intervertebral disc NOS
**V34**	**Revisional lumbar discectomy**	
V341	Revis laminec exc lumbar disc	Revisional laminectomy excision of lumbar intervert disc
V342	Revis fenestr exc lumbar disc	Revisional fenestration excision of lumbar intervert disc
V343	Rvsn ant exc lmbr disc+fusion	Revisional anterior excision of lumbar disc and fusion

V344	Rvsn ant excisn lmbr disc NEC	Revisional anterior excision of lumbar disc NEC
V345	Rvs ant excs lmbr dsc+pst fusn	Revisional anterior excision of lumbar disc and post fusion
V346	Rev ant excs lmbrdsc+pst instr	Revisional anterior excision lumbar disc + post instrument
V347	Rvsnl lmbr microdiscectomy	Revisional lumbar microdiscectomy
V348	Rvsnl post excsn lmbr disc	Revisional posterior excision of lumbar disc
V349	Revision lumbar discectomy NOS	Revisional excision of lumbar intervertebral disc NOS
**V35**	**Excis intervertebral disc NEC**	**Excision of intervertebral disc NEC**
V351	Primary excision IV disc NEC	Primary excision of intervertebral disc NEC
V352	Revision excision IV disc NEC	Revisional excision of intervertebral disc NEC
V358	Percutaneous discectomy	
V359	Excision intervertebr disc NOS	Excision of intervertebral disc NOS
V359/Y081	Laser discectomy	
**V37**	**Prim fusion cervical spine jnt**	**Primary fusion of joint of cervical spine**
V371	Brooks fuse atlantoaxial joint	Brooks fusion of atlantoaxial joint
V372	Post fusion joint cx spine NEC	Posterior fusion of joint of cervical spine NEC
V373	Transoral fuse atlantoax joint	Transoral fusion of atlantoaxial joint
V374	Fusion of atlantooccipital jnt	Fusion of atlantooccipital joint
V378	Fusion atlantoaxial joint NEC	Fusion of atlantoaxial joint NEC
V379	Cervic spine joint fusion NOS	Fusion of joint of cervical spine NOS
**V38**	**Oth prim fusion joint lumb sp**	**Other primary fusion of joint of lumbar spine**
V381	Prmry post fusion thorac spine	Primary posterior fusion of thoracic spine
V382	Prmry post fusn lmbr spine	Primary posterior fusion of lumbar spine
V383	Wiltse posterior fusion spine	Wiltse posterior fusion of spine
V384	Pry intrtrns fusn lmbr spn NEC	Primary intertransverse fusion lumbar spine NEC
V388	Primrposterolat fusn lmbr spn	Primary posterolateral fusion lumbar spine

V389	Prim fusion lumbar spine NOS	Primary fusion of joint of lumbar spine NOS
**V39**	**Other revision lumbar fusion**	**Other revisional fusion of joint of lumbar spine**
V391	Revisnal fusion cerv spine	Revisional fusion of cervical spine
V392	Revis post fusion thorac spine	Revisional posterior fusion of joint of thoracic spine
V393	Rvsn post fusn intrlam lmbr sp	Revision posterior interlaminar fusion of lumbar spine
V394	Revis post lumbar fusion NEC	Revisional posterior fusion of joint of lumbar spine NEC
V395	Rvs intrtrnsv fsn lmbr spn NEC	Revision intertransverse fusion of lumbar spine NEC
V398	Rvsn posterolat fusn lmbr spne	Revision posterolateral fusion lumbar spine
V399	Revision of lumbar fusion NOS	
**V41**	**Crct spn defrm+instrmntn**	**Correction of spinal deformity and instrumentation**
V411	Knodt spinal distraction rod	Posterior attachment Knodt spinal distraction rod to spine
V412	Crct spn defrm+ant instrmntn	Correction of spinal deformity and anterior instrumentation
V413	Removal Crctal spn instrmntn	Removal correctional spinal instrumentation
V418	Crct spn dfrm+instr+ped fxn sy	Crctn spn deform+instrumnt with pedicular fixation system
V419	Instrumental cor def spine NOS	Instrumental correction deformity of spine NOS
**V42**	**Other corr deformity spine**	**Other correction of deformity of spine**
V421	Excision of rib hump	
V422	Epiphysiodesis of spine	Epiphysiodesis of spine - deformity correction
V423	Antrolat release spn defrm+gft	Anterolateral release of spinal deformity and graft
V428	Other corr deformity spine OS	Other specified correction of deformity of spine
V429	Other corr deformity spine NOS	Correction of deformity of spine NOS
**V43**	**Extirpation spine lesion NEC**	**Extirpation of lesion of spine NEC**
V431	Excis lesion cervical vertebra	Excision of lesion of cervical vertebra
V432	Excis lesion thoracic vertebra	Excision of lesion of thoracic vertebra
V433	Excis lesion lumbar vertebra	Excision of lesion of lumbar vertebra

V438	Gill excis spondylolisthesis	Gill excision of spondylolisthesis
V439	Excision lesion of spine NEC	Excision of lesion of spine NEC
**V44**	**Spine fracture decompression**	**Decompression of fracture of spine**
V441	Complex decompress # spine	Complex decompression of fracture of spine
V442	Anterior decompression # spine	Anterior decompression of fracture of spine
V443	Posterior decompress # spine	Posterior decompression of fracture of spine
V448	Spine fracture decompress OS	Other specified decompression of fracture of spine
V449	Spine fracture decompress NOS	Decompression of fracture of spine NOS
**V45**	**Other spine fracture reduction**	**Other reduction of fracture of spine**
V451	Open reduct exc facet # spine	Open reduction of fracture of spine & excis facet of spine
V452	Open reduction # spine NEC	Open reduction of fracture of spine NEC
V453	Manipulative reduction # spine	Manipulative reduction of fracture of spine
V458	Spinal extension traction #	Spinal extension traction for fracture of spine
V459	Other spine fracture reduc NOS	Other reduction of fracture of spine NOS
**V46**	**Fixation of fracture of spine**	
V461	Pry opn red spn #+int fix+plte	Primary open reduc spinal fracture+internal fix+plate
V462	Fixat # spine Harrington rod	Fixation of fracture of spine using Harrington rod
V463	Pry opn red spn #+int fix+wire	Primary open reduc spinal fracture+internal fix+wire
V464	Halo skull traction # spine	Halo skull traction for fracture of spine
V468	Pry op rd sp #+int fix+seg wre	Primary open reduc spinal #+intern fix+segmental wire system
V469	Fixation spine fracture NOS	Fixation of fracture of spine NOS
**V52**	**Other intervertebral disc ops**	**Other operations on intervertebral disc**
V521	Enzyme destruct intervert disc	Enzyme destruction of intervertebral disc
V522	Destruction of disc NEC	Destruction of intervertebral disc NEC

V523	Discography intervert disc	Discography of intervertebral disc
V524	Prim ant/lat biops thorac disc	Primary anterolateral biopsy of thoracic intervertebral disc
V528	Other intervert disc op OS	Other specified operation on intervertebral disc
V529	Other intervert disc op NOS	Operation on intervertebral disc NOS
**V54**	**Other ops on spine & vertebra**	**Other ops on spine and vertebra**
V541	Transoral excisn odontoid peg	Transoral excision of odontoid peg
V542	Graft of bone to spine NEC	
V543	Osteotomy of spine NEC	
V544	Injection into paraspinal area	
V548	Primary thoracic spine op	Primary operation on thoracic spine
V549	Primary thoracic spine op NOS	Primary operation on thoracic spine NOS
**W01**	**Complex reconstruction thumb**	**Complex reconstruction of thumb**
**W01-W05**	**Complex reconstruct hand+foot**	**Complex reconstruction operations on hand and foot**
**W01-W92**	**Other bone & joint operations**	**Other bone and joint operations**
W011	Microvasc transf toe to thumb	Microvascular transfer of toe to thumb
W012	Pollicisation of finger	
W013	Thmb rcn usng bne grft+skn flp	Thumb reconstruction using bone graft and skin flap
W014	Thmb recon usng bne lngth proc	Thumb reconstruction using bone lengthening procedure
W015	Opponensplasty thumb	
W018	Free phalangeal transfer thumb	Free phalangeal transfer to thumb
W019	Complex reconstruct thumb NOS	Complex reconstruction of thumb NOS
**W02**	**Other complex reconstr of hand**	**Other complex reconstruction of hand**
W021	Proximal row carpectomy	
W022	Metacarpal supp op on carpus	Metacarpal support operation on carpus
W023	Multiple jnt reconstr hand NEC	Multiple joint reconstruction of hand NEC

W024	Cmplx soft tiss recons hnd NEC	Complex soft tissue reconstruction in hand NEC
W028	Reconstruction hand local flap	Reconstruction of hand with local flap
W029	Other complex reconst hand NOS	Other complex reconstruction of hand NOS
**W03**	**Complex reconstr of forefoot**	**Complex reconstruction of forefoot**
W031	Kessel reconstruction forefoot	Kessel reconstruction of forefoot
W032	Helal metatarsal osteotomy	
W033	Total correction of claw toe	
W034	Robert Jones proced great toe	Robert Jones procedure great toe
W035	Fusion joints mid & forefoot	Localised fusion of joints of midfoot and forefoot
W038	Correction of metatarsus varus	
W039	Complex reconstr forefoot NOS	Complex reconstruction of forefoot NOS
**W04**	**Complex reconstr of hindfoot**	**Complex reconstruction of hindfoot**
W041	Local fusion hindfoot joints	Localised fusion of joints of hindfoot
W042	Dunn triple fusion of foot	
W043	Goldthwait hindfoot stabilise	Goldthwait stabilisation of hindfoot
W044	Muscle strip from os calcis	Stripping of muscle from os calcis
W045	Exc lat wedge & fusion os calc	Rel medial soft tiss hindfoot & exc lat wedge fusion os calc
W048	Articular fusion subtalar jnt	Articular fusion subtalar joint
W049	Complex reconstr hindfoot NOS	Complex reconstruction of hindfoot NOS
**W05**	**Prosthetic replacement of bone**	
W051	Articul prosthet replace bone	Articulated prosthetic replacement of bone
W058	Prosthet replacement bone OS	Other specified prosthetic replacement of bone
W059	Prosthet replacement bone NOS	Prosthetic replacement of bone NOS
**W06**	**Excision of entire bone**	
W06-W36	Bone operations	

W061	Total excision of cervical rib	
W062	Total excision of rib NEC	
W063	Total excision of patella	
W064	Total excision of sesamoid NEC	Total excision of sesamoid bone NEC
W065	Talectomy	
W066	Total excision of coccyx	
W068	Total excision of bone OS	Other specified total excision of bone
W069	Ostectomy NEC	
W069+W059	Tot excis & prosthet rep bone bone	Total excision of bone and prosthetic replacement for bone
**W07**	**Excision of ectopic bone**	
W071	Excision of cross union bone	Excision of cross union of bone
W072	Excis periartic ectopic bone	Excision of periarticular ectopic bone
W073	Excis intramusc ectopic bone	Excision of intramuscular ectopic bone
W078	Excision of ectopic bone OS	Other specified excision of ectopic bone
W079	Excision of ectopic bone NOS	
**W08**	**Other excision of bone**	
W081	Excision of tuberosity of bone	
W082	Excision of overgrowth of bone	
W083	Excision of bony excrescence	Excision of excrescence of bone
W084	Excision of fragment of bone	
W085	Excision metatarsal head NEC	Excision of head of metatarsal bone NEC
W085+Z814	Rttr cuff dcomp-opn acrmplsty	Rotator cuff decompression - open acromioplasty
W088	Excision of synostosis	
W089	Other excision of bone NOS	Excision of bone NOS
W089+W051	Excis bone & art pros rep NEC NEC	Excision of bone and articulated prosthetic replacement NEC

W089+W059	Excis bone & prosth repl NEC	Excision of bone and prosthetic replacement NEC
W089+W329	Excis bone & bone graft HFQ qualified	Excision of bone and bone graft however further
**W09**	**Extirpation of lesion of bone**	
W091	Excision of lesion of bone	
W092	Curett bone lesion & graft HFQ	Curettage of lesion of bone and graft HFQ
W093	Curettage lesion of bone NEC	Curettage of lesion of bone NEC
W094	Destructn lesion of bone NEC	Destruction of lesion of bone NEC
W098	Cryoablation of bone lesion	
W099	Extirpation bone lesion NOS	Extirpation of lesion of bone NOS
**W10**	**Open osteoclasis**	
W101	Op osteocl ang cor int fix HFQ HFQ	Open osteoclasis, angular correction & internal fixation
W102	Op osteocl ang cor ext fix HFQ HFQ	Open osteoclasis angular correction & external fixation
W103	Angulatory osteotomy NEC	
W104	Open osteoclasis & int fix NEC	Open osteoclasis and internal fixation NEC
W105	Open osteoclasis+ext fxtn NEC	Open osteoclasis and external fixation NEC
W108	Rotational osteotomy	
W109	Open surgical fracture NOS	Open surgical fracture of bone NOS
**W11**	**Other surgical bone fracture**	**Other surgical fracture of bone**
W111	Closed osteoclasis	
W118	Other surg fracture of bone OS bone	Other specified other surgical fracture of
W119	Other surg fracture bone NOS	Other surgical fracture of bone NOS
**W12**	**Angulation periartic osteotomy**	**Angulation periarticular osteotomy**
W121	Bios ang peri ost int fix HFQ HFQ	Biosseus angulation periarticul osteotomy & int fixation
W122	Ang peri osteot & int fix NEC NEC	Angulation periarticular osteotomy and internal fixation
W123	Bios ang peri ost ext fix HFQ HFQ	Biosseus angulation periarticul osteotomy & ext fixation

W124	Ang peri osteot & ext fix NEC	Angulation periarticular osteotomy and external fixation NEC
W125	Biosseus ang peri osteot NEC	Biosseus angulation periarticular osteotomy NEC
W128	Akin's osteotomy	
W129	Angulat periartic osteot NOS	Angulation periarticular division of bone NOS
**W13**	**Other periarticular osteotomy**	
W131	Rotation periarticular osteot	Rotation periarticular osteotomy
W132	Displacement osteotomy	
W133	Cuneiform osteotomy	
W138	Other periarticular osteot OS	Other specified other periarticular division of bone
W139	Other periarticular osteot NOS	Other periarticular division of bone NOS
**W14**	**Diaphyseal division of bone**	
W141	Ang diaph osteot & int fix HFQ	Angulation diaphyseal osteotomy and internal fixation HFQ
W142	Ang diaph osteot & ext fix HFQ	Angulation diaphyseal osteotomy and external fixation HFQ
W143	Angulation diaphys osteot NEC	Angulation diaphyseal osteotomy NEC
W144	Rotat diaph ost & int fix HFQ	Rotation diaphyseal osteotomy and internal fixation HFQ
W145	Rotat diaph ost & ext fix HFQ	Rotation diaphyseal osteotomy and external fixation HFQ
W146	Rotation diaphys osteotomy NEC	Rotation diaphyseal osteotomy NEC
W148	Diaphyseal division of bone OS	Other specified diaphyseal division of bone
W149	Diaphyseal division bone NOS	Diaphyseal division of bone NOS
**W15**	**Osteotomy of bone of foot**	
W151	Mitchell hallux valgus osteot	Mitchell osteotomy for hallux valgus
W152	Golden hallux valgus operation	Golden osteotomy base 1st metatarsal bone for hallux valgus
W153	Hallux valgus osteotomy NEC	
W154	Osteotomy head metatarsal	Osteotomy of head of metatarsal
W155	Osteotomy of midfoot tarsal	

W158	Oth spec osteotomy foot bone	Other specified osteotomy of bone of foot
W159	Osteotomy of bone of foot NOS	
**W16**	**Other division of bone**	
W161	Multip osteot & int fix HFQ	Multiple osteotomy and internal fixation HFQ
W162	Multip osteot & ext fix HFQ	Multiple osteotomy and external fixation HFQ
W163	Multiple osteotomy NEC	
W164	Intn fixation of osteotomy NEC	Internal fixation of osteotomy NEC
W165	Extn fixation of osteotomy NEC	External fixation of osteotomy NEC
W168	Other division of bone OS	Other specified other division of bone
W169	Osteotomy NEC	

Supplementary Table S4: SAIL Databank sources for cohort data

	Rheumatology clinic data	Primary care data	Secondary care data	Office National Statistics Death Dataset
Gender		✓		
Age		✓		
BMI		✓		
Social deprivation quintile		✓		
Smoker		✓		
Alcohol drinker		✓		
RA codes	✓	✓		
AS codes	✓	✓		
PsA codes	✓	✓		
DMARDs		✓		
Biologic agents	✓			
Rheumatology clinic location	✓			
GP visit counts		✓		
Hyperlipidemia		✓		
Hypertension		✓		
Diabetes		✓		
Cardiovascular disease		✓		
Hospitalised with serious infections			✓	
Orthopaedic surgery			✓	
Disability payments		✓		
Sick notes issued		✓		
Date of death				✓

Supplementary Table S5: Univariate analysis for inclusion of significant candidate variables associated with commencing biologic treatment from diagnosis in AS patients to be incorporated into a cox proportional hazard model.

	Hazard ratio	95% CI
Female	1.15	0.83 to 1.59
Age at diagnosis	0.99	0.98 to 0.99*
Social deprivation quintile	0.96	0.87 to 1.07
BMI	1.02	0.99 to 1.05
Alcohol drinker	1.06	0.69 to 1.63
Smoker	0.88	0.56 to 1.40
Orthopaedic surgery	0.89	0.22 to 3.60
Hospitalised for serious infections	1.26	0.86 to 1.83
Disease activity score (DAS 28)	1.00	0.63 to 1.59
Existing diabetes	1.35	0.88 to 2.10
Existing cardiovascular disease	0.98	0.57 to 1.70
Existing hyperlipidemia	1.03	0.58 to 1.80
Existing hypertension	1.00	0.72 to 1.39
Number of NSAIDs pre-biologic	1.24	1.20 to 1.28*
Number of DMARDs pre-biologic	1.48	1.39 to 1.57*
DLA/PIP pre-biologic	5.91*	4.39 to 7.95*
Sick-notes issued pre-biologic	3.81	2.82 to 5.14*
GP visits pre-diagnosis	1.00	1.00 to 1.00
GP visits post-diagnosis	1.00	0.99 to 1.00
GP visits pre-biologic	1.00	1.0 o 1.00

**p<0.05*

Final Model (factors that remained significant):

Sick-notes issued pre-biologic; HR 1.60 (1.14 to 2.24)*

DLA/PIP pre-biologic; 4.26 (3.02 to 6.00)*

Supplementary Table S6: Univariate analysis for inclusion of significant candidate variables associated with commencing biologic treatment from diagnosis in PsA patients to be incorporated into a cox proportional hazard model.

	Hazard ratio	95% CI
Female	0.86	0.58 to 1.29
Age at diagnosis	0.98	0.97 to 0.99
Social deprivation quintile	1.13	0.99 to 1.30
BMI	1.01	0.98 to 1.04
Alcohol drinker	3.41	1.25 to 9.29*
Smoker	0.91	0.44 to 1.88
Orthopaedic surgery	0.48	0.07 to 3.48
Hospitalised for serious infections	1.14	0.66 to 1.95
Disease activity score (DAS 28)	1.01	0.82 to 1.25
Existing diabetes	1.04	0.56 to 2.0
Existing cardiovascular disease	0.68	0.30 to 1.55
Existing hyperlipidemia	0.93	0.45 to 1.91
Existing hypertension	0.96	0.61 to 1.50
Number of NSAIDs pre-biologic	1.51	1.44 to 1.57*
Number of DMARDs pre-biologic	2.56	2.35 to 2.78*
DLA/PIP pre-biologic	62.57	40.23 to 97.30*
Sick-notes issued pre-biologic	29.60	18.41 to 47.59*
GP visits pre-diagnosis	1.00	1.00 to 1.00
GP visits post-diagnosis	1.00	1.00 to 1.00
GP visits pre-biologic	1.00	1.00 to 1.01

**p<0.05*

Final Model (factors that remained significant):

Number of DMARDs pre-biologic; HR: 2.21 (1.87 to 2.60)*

DLA/PIP pre-biologic; HR 2.55 (1.15 to 5.66)

Supplementary Table S7: Univariate analysis for inclusion of significant candidate variables associated with biologic treatment failure in AS patients to be incorporated into a cox proportional hazard model.

	Hazard ratio	95% CI
Female	1.49	0.91 to 2.45
Age at diagnosis	1.00	0.98 to 1.02
Social deprivation quintile	1.05	0.89 to 1.24
BMI	1.05	1.01 to 1.10*
Alcohol drinker	0.87	0.45 to 1.71
Smoker	0.68	0.28 to 1.70
Orthopaedic surgery	3.00	0.94 to 9.53
Hospitalised for serious infections	1.15	0.63 to 2.09
Disease activity score (DAS 28)	2.46	0.75 to 8.06
Existing diabetes	0.92	0.42 to 2.02
Existing cardiovascular disease	1.07	0.46 to 2.47
Existing hyperlipidemia	0.45	0.11 to 1.84
Existing hypertension	1.11	0.65 to 1.89
Number of NSAIDs pre-biologic	1.02	0.94 to 1.10
Number of DMARDs pre-biologic	1.00	0.88 to 1.15
Biologic duration	1.52	1.41 to 1.64*
DLA/PIP pre-biologic	0.90	0.56 to 1.46
DLA/PIP post-biologic	1.53	0.87 to 2.68
Sick-notes issued pre-biologic	1.01	0.62 to 1.66
Sick-notes issued post-biologic	0.87	0.47 to 1.63
GP visits pre-diagnosis	1.00	1.00 to 1.00
GP visits post-diagnosis	1.00	1.00 to 1.00
GP visits pre-biologic	1.00	1.00 to 1.00
GP visits post-biologic	1.01	1.00 to 1.01

$^*p<0.05$

Final Model (factors that remained significant):

Biologic duration; HR: 1.51 (1.39 to 1.64)*

Supplementary Table S8: Univariate analysis for inclusion of significant candidate variables associated with biologic treatment failure in PsA patients to be incorporated into a cox proportional hazard model.

	Hazard ratio	95% CI
Female	1.28	0.84 to 1.96
Age at diagnosis	0.99	0.97 to 1.00
Social deprivation quintile	1.05	0.92 to 1.20
BMI	1.02	0.99 to 1.05
Alcohol drinker	1.84	0.74 to 4.54
Smoker	1.59	0.85 to 2.99
Orthopaedic surgery	2.00	0.73 to 5.45
Hospitalised for serious infections	1.21	0.73 to 4.45
Disease activity score (DAS 28)	1.00	0.95 to 1.06
Existing diabetes	0.81	0.39 to 1.67
Existing cardiovascular disease	0.39	0.10 to 1.58
Existing hyperlipidemia	0.95	0.42 to 2.18
Existing hypertension	1.50	0.97 to 2.33
Number of NSAIDs pre-biologic	0.97	0.84 to 1.13
Number of DMARDs pre-biologic	0.83	0.47 to 1.46
Biologic duration	1.32	1.24 to 1.42*
DLA/PIP pre-biologic	1.01	0.65 to 1.55
DLA/PIP post-biologic	1.51	0.85 to 2.67
Sick-notes issued pre-biologic	0.79	0.50 to 1.26
Sick-notes issued post-biologic	1.33	0.83 to 2.15
GP visits pre-diagnosis	1.00	1.00 to 1.00
GP visits post-diagnosis	1.00	1.00 to 1.00
GP visits pre-biologic	1.00	1.00 to 1.00
GP visits post-biologic	1.00	1.00 to 1.01

$*p<0.05$

Final Model (factors that remained significant):

Biologic duration; HR 1.32 (1.24 to 1.42)*

Supplementary Figure S1: Incidence of biologic initiation in AS patients by year

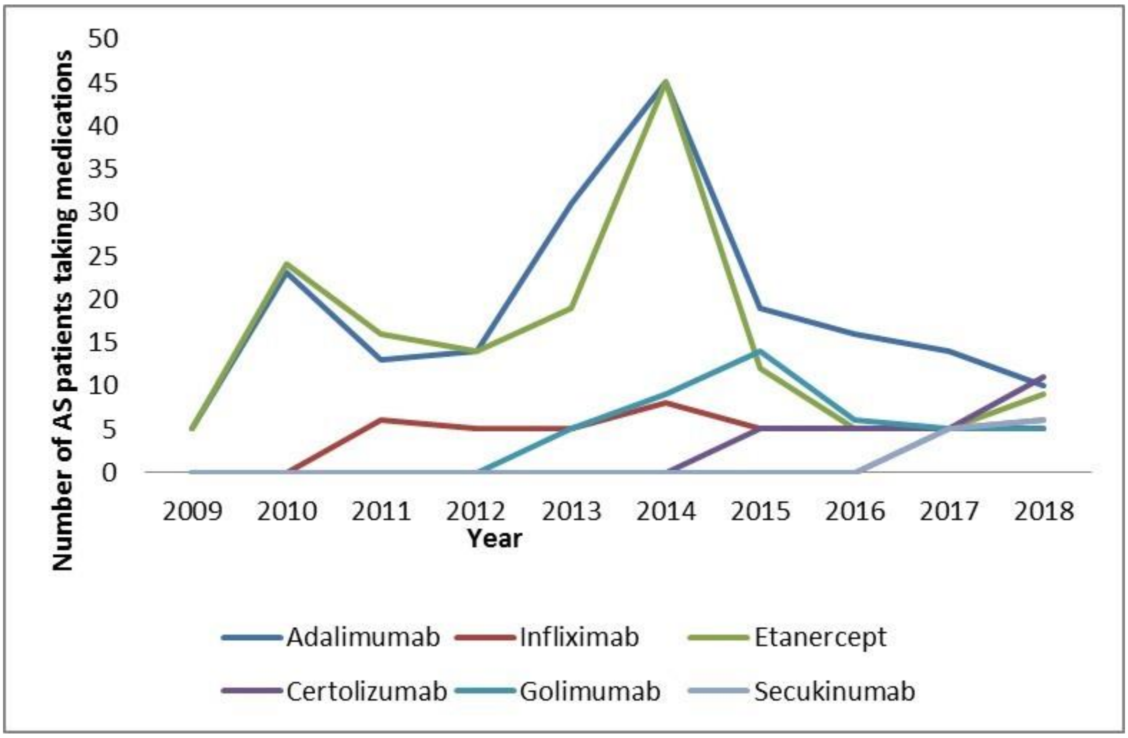

Supplementary Figure S2: Incidence of biologic initiation in PsA patients by year

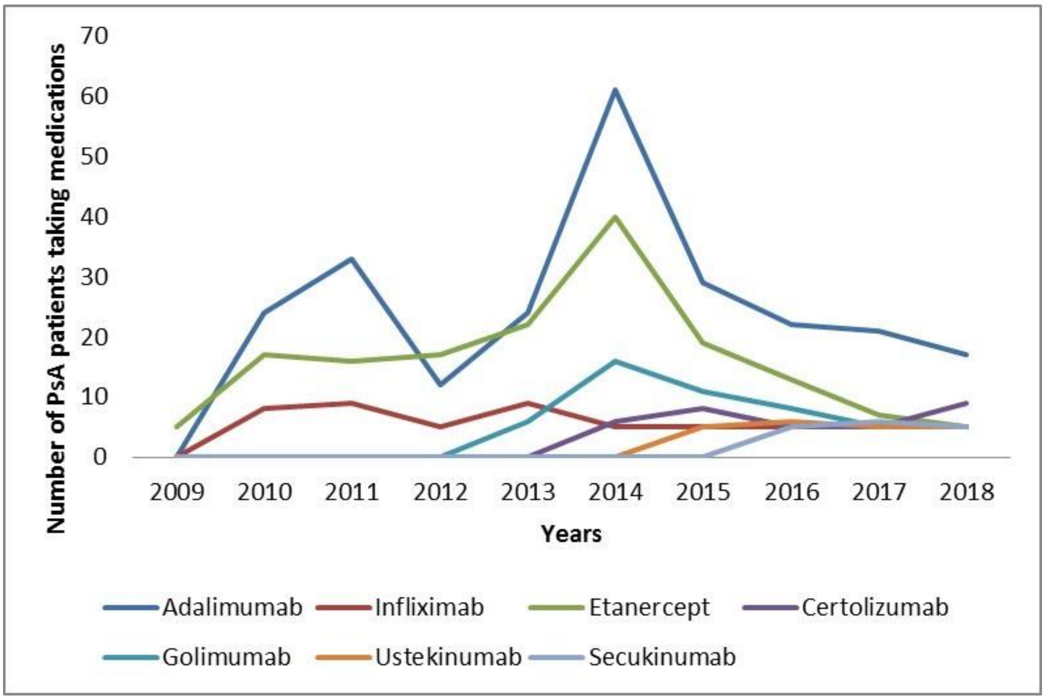

Supplement: rkab042_Supplementary_Data [file rkab042_supplementary_data.pdf]
